# ProfhEX: Empowering Early Drug Discovery with Machine Learning-Based Target Profiling and Liability Prediction

**DOI:** 10.1021/acs.jcim.5c02250

**Published:** 2025-12-08

**Authors:** Filippo Lunghini, Carmen Cerchia, Anna Fava, Vincenzo Pisapia, Francesco Sacco, Andrea Rosario Beccari

**Affiliations:** † EXSCALATE, 18798 Dompé Farmaceutici SpA, Via Tommaso de Amicis 95, 80123 Naples, Italy; ‡ Department of Pharmacy, 9307 University of Naples “Federico II”, Via D. Montesano 49, 80131, Napoli, Italy; § Professional Service Department, SAS Institute, Via Darwin 20/22, 20143 Milan, Italy

## Abstract

The drug discovery process is inherently lengthy, complex, and costly, with high attrition rates driven by safety concerns, limited efficacy, and regulatory barriers. AI-driven computational methods have become crucial in accelerating this process by enabling the prediction of molecular activities, identification of off-target interactions, and prioritization of candidates for drug repurposing. However, existing ligand-based prediction tools often suffer from limited data coverage, narrow target scopes, and usability challenges. Here, we present an enhanced version of ProfhEX, a scalable and user-friendly platform designed for comprehensive drug–target activity profiling. The updated platform features 969 predictive models spanning 693 human targets, trained on over 5 million curated bioactivity data points. ProfhEX demonstrates high predictive accuracy in prospective real-world scenarios and surpasses state-of-the-art tools in primary target prediction benchmarks. ProfhEX represents one of the largest and most accurate platforms for compound–target prediction, supporting early stage drug discovery and enhancing target liability assessment.

## Graphical abstract

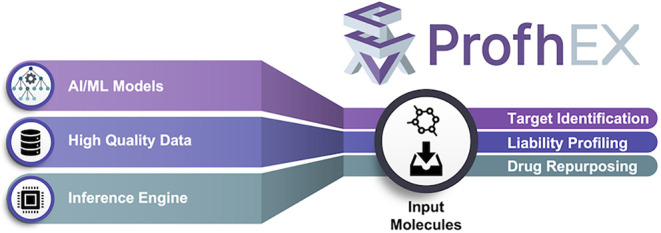

## Introduction

The drug discovery process is lengthy, complex, and costly, with high attrition rates due to safety risks, insufficient efficacy, and regulatory hurdles.^1,2^ Computational approaches, particularly target and bioactivity prediction, have become key to accelerating discovery. The use of machine learning (ML) and artificial intelligence (AI) has expanded rapidly,^3^ enabling modeling and evaluation of molecular interactions.^4,5^ In particular, identifying protein targets for small molecules supports inhibitor design, off-target estimation, polypharmacology analysis,^6^ and drug repurposing.^7,8^

Among the methods for predicting compound potency, quantitative structure–activity relationship (QSAR) models^9^ remain central in medicinal chemistry, including both linear approaches for analog series and modern ML for diverse compounds.^10^

Recent advances feature ML-based scoring functions and deep learning (DL) models that combine ligand and protein information.^11,12^ These tools predict binding, pose, and affinity, relying on curated structural data sets. However, most ML scoring functions are standalone, unlike docking-integrated classical ones. DL models achieve strong results but remain task-specific, hindered by limited structure–affinity data. Integrating physics-informed components and data augmentation, as in PIGNet2 ^13^ and DeepRLI^14^ has improved generalization. For a more detailed discussion, we refer readers to recent comprehensive reviews.^11,12,15^

AI-based methods often require expertise and resources, limiting accessibility. Several web-based tools aim to address this by providing ligand-based models for target prediction: notable examples are the Similarity ensemble approach (SEA),^16^ HitPick,^17^ PASS Targets,^18^ and the updated version of SwissTargetPrediction.^19^ Recently published Web servers employ ML models rather than overall structural similarity, such as TargetNet,^20^ SuperPred,^21^ CoDDPred,^22^ and AmIActive.^23^ Interestingly, some applications were developed to predict ligand’s activity for specific targets, such as serotonin receptors,^24^ epigenetic targets,^25^ or targets involved in Alzheimer’s disease.^26^ On the other hand, Web servers built on structure-based methods have also been reported, aimed at the identification of ligand-target interaction predictions.^27,28^ Yet, most methods suffer from limited training data, restricted target coverage, protein-family bias, small input size, or discontinued access.

We recently introduced ProfhEX,^29^ covering 46 liability-related targets across seven organ systems. These targets were selected from those routinely assessed in vitro by pharmaceutical companies for liability profiling.^30^ Here, we present a major expansion: 969 models for 693 human targets, trained on >5 million bioactivity data points from updated sources. This release is, to our knowledge, the largest platform for compound–target activity prediction. ProfhEX combines validated accuracy with an intuitive interface, supporting discovery of new agents, liability detection, and repurposing opportunities. In addition to the greatly increased scope, ProfhEX has been validated in multiple real-world application scenarios, including primary target identification, potency prediction, and enrichment factor evaluation. Unlike most servers, ProfhEX enables batch input of up to 100 SMILES, making it both powerful and user-friendly. The platform is freely accessible at: https://profhex.exscalate.eu/.

## Results and Discussion

### Overview of the Available Models

Below we provide an overview of the models and their targets. Figure 1 depicts the number of available models grouped into the main protein families. Each stacked histogram bar illustrates the repartition of the three activity values (EC50, IC50, *K*_i_) in the respective family group. As shown in Figure 1 and summarized in Table S1, the amount of available data varies substantially across protein families, reflecting both experimental focus and therapeutic interest. Enzymes and membrane receptors represent the largest portion of the chemical space, with individual models ranging from a few to several thousand compounds, highlighting their broad biochemical diversity and central role in drug discovery. GPCRs are well-studied due to their role in signaling, whereas kinases and proteases are also critical, often targeted in cancer and inflammatory diseases. The abundance of models for these proteins reflects their therapeutic relevance and available structural data, aiding in the development of precise in silico models for virtual screening. Ion channels, epigenetic regulators, and transcription factors have moderate data set sizes, typically ranging from hundreds to a few thousand entries, which is sufficient to train robust predictive models while capturing family specific structural and functional variability. Smaller families, such as adhesion proteins, auxiliary transporters, and secreted proteins, have data sets of only a few hundred compounds, reflecting either limited experimental characterization or specialized biological functions. There is a clear prevalence of antagonists/inhibitors over agonist models, mostly due to the therapeutic focus of drug discovery, where antagonists are commonly developed to inhibit overactive pathways associated with various diseases. Additionally, measuring IC50 values is generally more straightforward and reliable in experimental assays than measuring EC50 values for agonists,^31^ which require capturing receptor activation dynamics.^32^ A remarkable exception is represented by the GPCR family A, for which a higher number of models based on EC50 could be generated. One potential reason is that these receptors are key regulators of physiological processes, such as neurotransmission, hormone regulation, and sensory perception, where activating the receptor can restore or enhance normal cellular functions.^33,34^ Table S1 summarizes the average data set size and performance metrics of the ML models grouped by protein family, while Table S2 provides a detailed list of all models, along with target property statistics and cross-validation performance.

**Figure 1:**
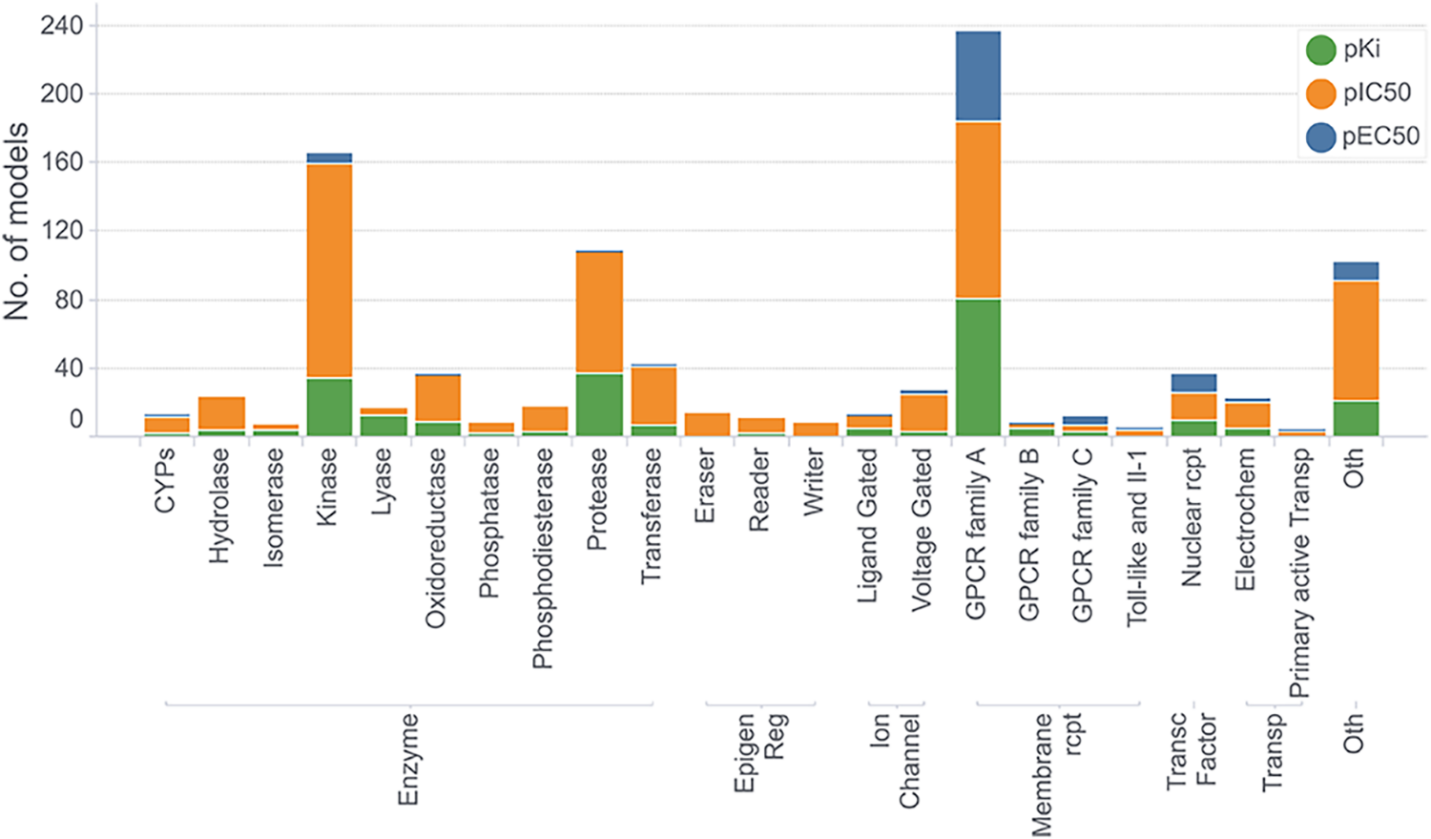
Distribution of ProfhEX models across the main protein families. The colors green, orange, and blue represent the models based on quantitative measures of pKi, pIC50, and pEC50, respectively.

We complemented the tabular data with both static and interactive visualizations of model performance. In the Figure S2, a bar chart reports the average *R* ^2^ (blue bars) and RMSE (red bars) across protein families. Predictive performance is generally stable (*R* ^2^ ≈ 0.65–0.80; RMSE ≈ 0.55–0.75) across most families. For example, Toll-like/IL-1 receptors and GPCR family B achieve high average *R* ^2^ (∼0.79–0.78) with data set sizes around 2000–6000 compounds, whereas CYP enzymes display lower *R* ^2^ (∼0.59) despite data sets of comparable size, reflecting intrinsic chemical and biological heterogeneity. Conversely, smaller families such as Aminoacyltransferases or ion channel subsets achieve high performance despite more limited data, suggesting that favorable SAR landscapes can compensate for restricted data set size. These observations confirm that data set size alone is not the primary determinant of model quality. To further enhance transparency and usability, we provide an interactive web-based visualization (available in our Zenodo repository, profhex_analysis.html) that allows users to sort protein families by *R* ^2^, RMSE, data set size, or family name, and to explore scatter plots of data set size versus performance.

### Models’ Performance

The automated modeling procedure generated over 1500 models, with 969 meeting the specified quality criteria. A key exclusion criterion was data set size, as approximately 30% of the target data sets contained fewer than one hundred compounds. Such a sample size is typically insufficient for creating reliable models, especially with highly diverse compounds, as it complicates the learning of robust structure–activity relationships and limits the models’ applicability for virtual screening. This indicates that several targets are significantly underrepresented in terms of data availability and chemical diversity and will benefit from the generation of new experimental data. Overall, ProfhEX models exhibited strong predictive power (Figure 2), with an average *R* ^2^ of 0.68 (SD = 0.1), an *R* value of 0.83 (SD = 0.06), an RMSE of 0.67 (SD = 0.14), and an enrichment value of 12.4 (SD = 4.7). The RMSE of the models is comparable to the variability of experimental affinity measurements.

**Figure 2:**
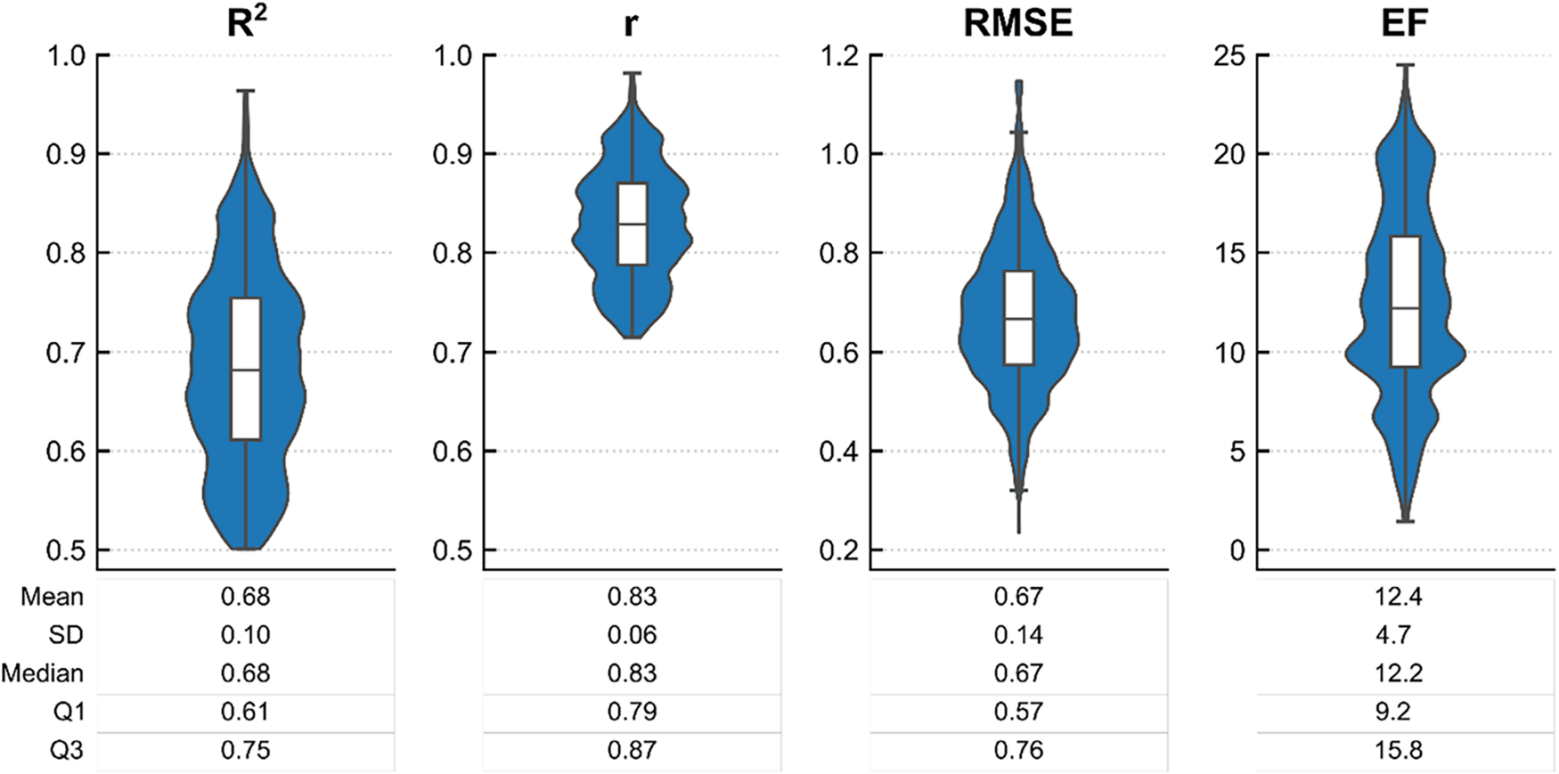
Box plots illustrating the distribution of ProfhEX models’ regression metrics. From left to right: determination coefficient (*R* ^2^), Pearson correlation coefficient (*r*), root mean squared error (RMSE), and enrichment factor (EF) at 5%.

Learning-curve analyses further indicated that model performance improves rapidly with increasing data set size but tends to plateau once several thousand data points are reached. This saturation is consistent with previous large-scale ChEMBL modeling efforts,^35,36^ where diminishing returns were observed beyond ∼10–20k compounds due to assay variability and chemical redundancy. In our case, additional data points contributed little to reducing the RMSE beyond the experimental uncertainty (≈0.5–0.7 log units), which represents a practical error floor.^37^ Importantly, however, enlarging the data sets still provided clear advantages: it broadened the applicability domain of the models, reduced the risk of overfitting to narrow chemical series, and improved the capacity to extrapolate toward novel scaffolds.

### Web Interface Overview

The platform has been designed to be both intuitive and comprehensive, making it accessible to expert and nonexpert users alike. Before launching predictions, users can explore the full set of available models through an interactive dashboard (Figure 3), which provides a tree map of protein families, summaries of the training data sets, and multiple performance indicators (RMSE, *R* ^2^, MAE) derived from cross-validation, external validation, and bootstrapping. For activity estimation, compounds are submitted as SMILES strings, either individually or in batches of up to 100 molecules, a feature rarely available in comparable web services. Users can then select protein families or activity types of interest (IC50, EC50, or *K*_i_), and results are returned in two complementary formats: a downloadable file for offline analysis and an interactive web report for immediate exploration. The web report integrates SMILES with two-dimensional (2D) structures, predicted activities, average activity values per compound (Average pACT), and two visualization tools: a hierarchical heatmap grouping targets into functional classes (e.g., Writer, Reader, Eraser) for rapid assessment of activity distribution, and a compound–target interaction heatmap with filtering options for potency thresholds or specific protein families. The downloadable CSV file (see Supporting Information for a sample output) provides a complete compound–target prediction table, with each row representing a molecule–target pair. Compounds are reported as standardized SMILES strings, while targets are identified by UniProt accession numbers and gene names. Predictions are expressed as binding affinity values, where higher scores indicate stronger interactions. To contextualize these predictions, each target is further classified within a two-level hierarchy derived from UniProt, first by broad protein family (e.g., ion channels) and then by functional subtype (e.g., voltage-gated channels).

**Figure 3:**
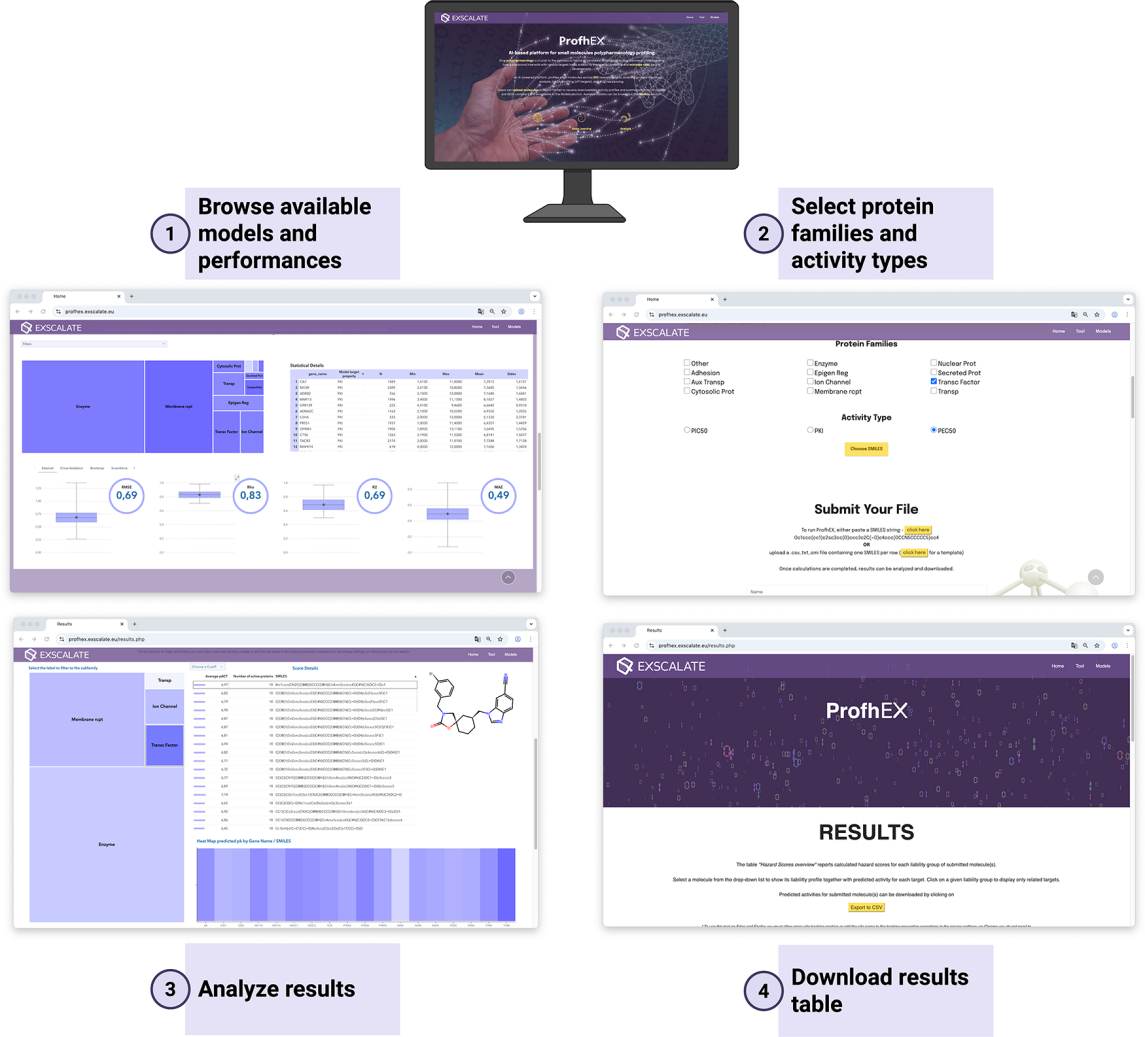
Overview of the ProfhEX web platform. The workflow illustrates the inference process. From the main page, users can browse existing models, select target proteins, and choose activity types. Once the calculation is complete, results can be explored and analyzed through the results report. Data export is also available for offline use. Created in BioRender.

### Case Studies

To further evaluate the predictive power of ProfhEX’s models and illustrate potential real-world applications, this section addresses four distinct use cases: (1) prospective evaluation of ProfhEX predictions for compounds from recent patents and scientific literature; (2) potency prediction for a set of compounds; (3) performance in primary target identification and (4) virtual screening performance evaluation on the LIT-PCBA benchmark data set (see Supporting Information Case study (4)).^38^ In all four cases, to ensure proper validation, we verified that none of the new compounds were included in ProfhEX models’ training set.

#### Prospective Evaluation of ProfhEX Predictions for Compounds from Recent Patents and Scientific Literature

For the case studies, ProfhEX models were trained on data available prior to 2023 to enable an unbiased, prospective evaluation on compounds reported in 2023–2024. Following validation, all models were retrained using the full ChEMBL, PubChem, and GOSTAR releases up to 2024, and the updated models are available on the ProfhEX web platform. Given the sparse nature of the data matrix, we included only those targets with at least 50 newly reported measurements to ensure robust performance evaluation. This filtering process yielded 7502 new compounds across 18 targets (Table S3; raw data available in our Zenodo repository, Case Study #1). The results are presented in Table 1 and visualized in the scatterplot of Figure 4.

**Figure 4:**
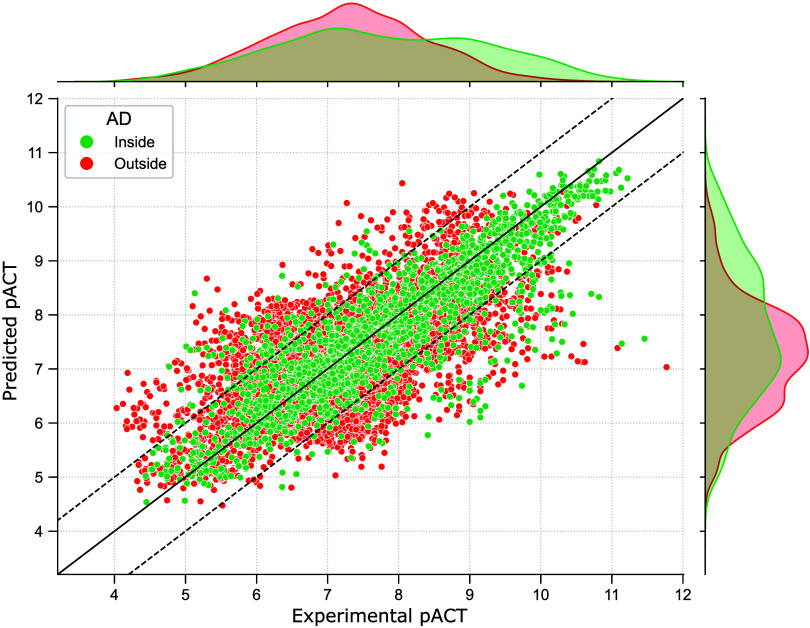
Scatterplot illustrating the results of the prospective evaluation of new compounds. Green and red dots represent data points inside and outside the Applicability Domain (AD), respectively. The *x*-axis corresponds to the observed pACT values, and the *y*-axis to the predicted pACT values, showcasing the distribution of performance across the data set. The solid line represents the line of identity bisector, while the dashed lines indicate the models’ standard error boundaries.

**Table 1:** Results from the Prospective Evaluation of New Compounds^a^

applicability domain	# of compounds	*r*	RMSE	MAE	MaxE
all data	7502	0.52 (0.24)	0.91 (0.40)	0.70 (0.29)	2.67 (0.82)
inside-only	1841	0.63 (0.26)	0.78 (0.38)	0.59 (0.28)	2.23 (0.86)

a The average performance across the 18 targets is reported, including the Pearson correlation coefficient (r), root mean squared error (RMSE), the mean absolute error (MAE) and the maximum absolute error (MaxE). Standard deviations are shown in parentheses.

Overall, the models demonstrated robust predictive power, with *r* = 0.52 – 0.63 and RMSE = 0.91 – 0.78 for all compounds and for those within the applicability domain, respectively. However, we observed a relatively modest determination coefficient (*R* ^2^ ≈ 0.3). This discrepancy stems from the well-known sensitivity of *R* ^2^ to the range and variance of the observed values in the validation set. Specifically, when the variance of the dependent variable is small, *R* ^2^ can appear low, even if the model accurately captures the relationship between predicted and observed values. As frequently reported in the literature, QSAR models tend to perform better when predicting compounds within their applicability domain.^39^ In our analysis, restricting predictions to this domain resulted in approximately a 20% improvement in accuracy. However, this gain in predictive reliability was accompanied by a notable trade-off: only about one-third of the total compound set fell within the applicability domain.

To evaluate potential information leakage between training and test sets, we computed pairwise Tanimoto similarities using ECFP4 fingerprints. Each test compound was compared against all training compounds, retaining the maximum similarity to its closest analogue. These values were then averaged to yield a global measure of overlap, with detailed results reported in Table S4. On average, test compounds exhibited moderate similarity to their nearest neighbors in the training set (mean maximum Tanimoto = 0.60, SD = 0.13), suggesting that a large fraction of the test molecules remained structurally distinct. Even at the 90th–95th percentile, similarity values plateaued around ∼0.8, suggesting that while a minority of test molecules have close analogues, the majority were more diverse. To further assess structural distinctiveness, we quantified scaffold overlap using two complementary definitions: wireframe scaffolds, which capture only the carbon skeleton, and Bemis–Murcko scaffolds, which offer a more restrictive definition of the core. Across all case studies, scaffold overlap remained relatively low on average. Wireframe overlap ranged from 0.11 to 0.84 (mean = 0.39, SD = 0.21), showing heterogeneous retention of generic skeletons. Bemis–Murcko overlap was consistently lower (range = 0.01–0.57, mean = 0.15, SD = 0.15), confirming that most chemical cores in the test sets were absent in the training data. Notable exceptions included LTA4H and TRPV3, which showed >30% Bemis–Murcko overlap and the highest average Tanimoto similarity (∼0.77). These targets also yielded the lowest prediction error (RMSE ∼ 0.38, less than a half-log unit deviation in potency estimation), reflecting the presence of closer structural analogues in the training data. Despite this, the overall benchmark remains nontrivial. For these 18 targets, all predictive models were based on gradient boosting. In summary, the limited overlap between training and test sets supports that predictive performance was not driven solely by close analogue memorization, reinforcing the robustness and generalizability of the benchmarking design

#### Potency Prediction for a Set of Compounds

To further assess the predictive performance of ProfhEX, we conducted an independent case study based on a manually curated data set from the scientific literature. Large public databases such as ChEMBL provide a valuable source of experimental bioactivity data, however they may contain inconsistencies or incomplete assay annotations, which can complicate model evaluation. To mitigate this issue, we selected a recent virtual screening study published in 2024 by Higgins et al.,^40^ which reported detailed experimental validation of newly tested compounds on a specific target. Again, we confirmed that none of the compounds in this study were present in the training set, ensuring a genuine blind prediction scenario. Higgins et al.^40^ identified novel inhibitors of human dihydroorotate dehydrogenase (DHODH, UniProt Q02127), a key target for treating cancer and autoimmune diseases. The authors reported 20 compounds with experimental IC50 values between 91 nM and 2.7 μM. The ProfhEX model showed very good predictive power for these 20 compounds, achieving a Pearson correlation of 0.66 and an RMSE of 0.63, which is comparable to the average model’s error (RMSE_avg_ = 0.70). Raw data are available in our Zenodo repository (Case Study #2). The only exception was the outlier compound 16, which was significantly underestimated by the model. It should be noted that this compound was flagged as out-of-applicability due to its chemical structure, which is significantly different from the compounds in the training set. As shown in Figure S3, the substructure of this compound highlighted in light blue is a chemical moiety that has never been captured in the training set. Interestingly, compound 16 showed inconclusive results in the cell viability assay. The authors reported that the lack of cellular activity may be due to its symmetrical structure and the presence of Schiff bases, which are chemically reactive and could have formed unexpected byproducts, potentially interfering with DHODH inhibition.^40^ These factors could explain the discrepancy between its biochemical potency and cellular effects.

#### Performance in Primary Target Identification

In this section, we evaluate the performance of our platform in identifying primary targets for a set of compounds and compare our results with several other target-prediction tools, such as SwissTargetPrediction,^41^ SuperPred,^21^ PASS Targets,^18^ CoDDPred,^22^ TargetNet,^20^ and AmIActive.^23^

Starting with the prospective data set of compounds from case study #1, we first selected those with at least nanomolar activity (i.e., pACT ≥ 7). From this subset, we then chose 10 chemically diverse (ECFP4 based) compounds per target, resulting in a total of 170 compounds spanning 17 targets (TRPV3 was excluded since no compounds met the required pACT ≥ 7 threshold). While this approach is not exhaustive, we believe it is robust enough to cover a representative portion of the chemical space, providing a fair and reliable evaluation across the tools. The complete list of selected compounds and their targets is reported in Table S5.

We provide in Table S6 a comparative analysis of ProfhEX against these tools, summarizing the underlying model types, training data sources, data set sizes, reported performance metrics, number of modeled targets, and key functional features.

For each compound, predictions were generated across all available ProfhEX models as well as with the other tools. Most of the analyzed tools do not support batch compound screening, which significantly limits their capabilities. Evaluation of target identification was based on whether the experimentally known primary target of each compound appeared within the top-N ranked predictions, measuring the accuracy of primary target recovery. The results are reported in Table 2. Raw data are available in our Zenodo repository (Case Study #3).

**Table 2:** Performance of Each Tool in Identifying Correct Primary Targets^a^

	top *N* accuracy (%)				
tool	*N* = 1	*N* = 5	*N* = 10	*N* = 15	*N* = 20
ProfhEX AD All	21	36	42	52	58
ProfhEX AD In	**54**	**85**	**91**	**95**	**98**
SwissTargetPrediction	5	10	10	11	12
SuperPred	5	9	13	15	15
PASS	0	2	10	22	32
CODD-Pred	22	31	34	35	36
TargetNet	0	2	6	10	15
AmIActive	3	15	23	28	35

a The table reports the average percentage of correct primary targets identified within the top-1, top-5, top-10, top-15, and top-20 predictions for 170 selected compounds. For ProfhEX, we report prediction for all compounds (AD All) and for only those inside the applicability domain (AD In).

ProfhEX demonstrated very good predictive performance in target identification, surpassing state-of-the-art platforms. SwissTargetPrediction and SuperPred demonstrated low predictive accuracy, with minimal gains observed as the number of top-ranked targets increased, suggesting limited predictive power. In contrast, PASS showed poor accuracy at lower thresholds (small N) but improved substantially with higher N, indicating a broader yet less precise prediction profile. CODD-Pred exhibited good performance, with steady but slower improvement compared to ProfhEX, which consistently outperformed all other methods. Notably, ProfhEX includes a higher number of targets than other platforms (969), making the baseline probability of correct predictions more challenging. In line with the findings from case study #1, the results highlight the substantial impact of applying the AD filter, which leads to more than a 2-fold increase in accuracy. To ensure a fairer comparison, we performed an additional evaluation for the other platforms. Since formal applicability domain (AD) definitions are not available for all platforms, and complete target lists are provided only for some, a target was considered in-domain if it appeared in the platform’s output list of predicted targets. The results, reported in Table S7, illustrate the trade-off between coverage and accuracy: restricting the evaluation to in-domain predictions increased accuracy while reducing overall coverage.

ProfhEX can not only identify the expected primary targets but also highlight additional plausible targets from a compound’s broader activity profile, providing a foundation for both drug repurposing and comprehensive liability assessment. The extended activity profiles support the identification of alternative therapeutic indications and potential safety liabilities. In this way, beyond assessing primary target recovery, the ranked predictions of secondary targets provide valuable insight into novel therapeutic opportunities and the broader polypharmacology of compounds.

## Conclusion

In this paper we presented an enhanced version of ProfhEX,^29^ a freely accessible platform for profiling the activity of small molecules across 693 human targets. ProfhEX provides a user-friendly resource for liability assessment, compound–target prediction, polypharmacology analysis, repurposing exploration, and virtual screening. The platform showed strong performance in multiple validations, including compounds from recent patents and literature, curated potency data sets, and primary target identification, where it outperformed other state-of-the-art tools. Nonetheless, accuracy depends on each model’s applicability domain, which limits coverage for underrepresented targets, and performance is influenced by data quality and quantity, with kinases and GPCRs generally outperforming smaller or heterogeneous families. This release focuses on human proteins; off-target and repurposing opportunities are provided in the results tables for user prioritization. Future updates will enhance visualization of secondary targets and integrate ADMET properties, metabolic pathway prediction, and uncertainty quantification. The platform is freely accessible at: https://profhex.exscalate.eu/. We believe that ProfhEX has the potential to accelerate drug discovery by enabling faster evaluation and optimization of compounds with desired target activity.

## Supplementary Material





## Data Availability

The ChEMBL database (https://www.ebi.ac.uk/chembl/) and PubChem (https://pubchem.ncbi.nlm.nih.gov) are public domain data resources. GOSTAR data sets were collected from the GOSTAR database (https://www.gostardb.com/). The models built in this study are freely available via an interface at https://profhex.exscalate.eu/. Noncommercially protected training set molecules, data preparation protocol, raw data from the case studies are available in our Zenodo repositories (https://zenodo.org/records/7665586 and https://zenodo.org/uploads/17073900).

## Notes

Detailed protocols for data curation, preprocessing, and model development, including training, validation, and deployment workflows (Supporting Methods). Additional figures and tables provide comprehensive analyses of model coverage, performance, and benchmarking: ProfhEX workflow, distribution of models across protein families, and performance metrics (Figures S1–S2 and Tables S1–S2); prospective validation results, including structural overlap analyses and external compound testing (Tables S3–S4 and Figure S3); compounds and targets used in case study 3 and benchmark comparisons with other drug–target prediction tools, including in-domain filtering (Tables S5–S7); virtual screening results on the LIT-PCBA data set (Case Study 4 and Table S8) (PDF)
Example of output file illustrating the compound–target prediction results (CSV)
