# Supplementary material for: ProfhEX: Empowering Early Drug Discovery with Machine Learning-Based Target Profiling and Liability Prediction
Source: J Chem Inf Model. 2025 Dec 8;65(24):13037–44. doi: 10.1021/acs.jcim.5c02250 (PMC12728921; doi:10.1021/acs.jcim.5c02250)
Supplement: Supplementary file 1 [file ci5c02250_si_001.pdf]

Supporting Information

ProfhEX: Empowering Early Drug Discovery with Machine Learning-Based Target Profiling and Liability Prediction

Filippo Lunghini[1*], Carmen Cerchia[2], Anna Fava[1], Vincenzo Pisapia[3], Francesco Sacco[3], Andrea Rosario Beccari[1*]

[1]EXSCALATE, Dompé Farmaceutici SpA, Via Tommaso de Amicis 95, 80123 Naples, Italy

[2]Department of Pharmacy, University of Naples “Federico II”, Via D. Montesano 49, 80131, Napoli, Italy

[3]Professional Service Department, SAS Institute, Via Darwin 20/22, 20143 Milan, Italy

*To whom correspondence should be addressed. Email: Andrea.Beccari@dompe.com. Correspondence may also be addressed to: Filippo.Lunghini@dompe.com

Supporting methods....3
Data preparation....3
Model development pipeline and MLOps framework....3
ProfhEX web interface implementation....4
Supporting figures and tables....5
Figure S1.....5
Table S1....6
Figure S2....7
Table S2....8
Table S3....38
Table S4....39
Figure S3....40
Table S5....41
Table S6....51
Table S7....55
Case study 4. Virtual screening performance evaluation on the LIT-PCBA benchmark dataset....56
Table S8....57
Supporting References....58

Supporting methods

Data preparation

Experimental activity data were collected from multiple public and commercial sources, including ChEMBL, PubChem, and Excelra's GOSTAR database up to release v.2024. For each target, all available human-derived activity measurements were retrieved using UniProt accession IDs, which served as the primary reference to ensure unambiguous mapping of each record to its corresponding protein. Only IC50, EC50 or K_i measurement values were retained, while censored measurements (e.g., ">" or "<") were excluded. Target standardization and grouping were carried out using UniProt IDs in combination with the activity measurement type, which reflects the experimental mode of action (e.g., antagonist, binder or agonist). This composite key enabled consistent extraction and merging of activity data across sources while avoiding inconsistencies in trained models' nomenclature. Higher order protein family assignments were derived directly from UniProt hierarchical annotations, providing a reproducible mapping of individual proteins into broader functional families. This scheme supports automated downstream model operations (MLOps) and mimics real-world applications such as virtual screening and polypharmacology prediction, where inference is typically anchored on a single protein target and mode of action.

When multiple activity measurements existed for the same compound, the median pACTIVITY (pACT) value was calculated, normalized to the negative logarithmic molar scale. Compound structures, originally represented as SMILES strings, were standardized following conventional rules. De-duplication was based on standardized SMILES matching.

To construct the molecular feature space, we combined RDKit-derived physicochemical descriptors with circular molecular fingerprints. Specifically, ECFP4 and FCFP4 count-based fingerprints (radius 4, 1024 bits each) replaced the ECFP6/FCFP6 fingerprints used in the previous ProfhEX release, motivated by internal benchmarking on the expanded panel of targets where ECFP4 exhibited slightly improved generalization. The physicochemical descriptor set was extended from 11 to 208 RDKit-calculated properties, capturing global molecular characteristics including topological, electronic, and spatial features. The resulting input vectors comprised 2256 features per compound, integrating local structural patterns and holistic physicochemical attributes. Feature computation and preprocessing were automated within SAS Viya 4.0, a distributed in-memory analytics platform optimized for handling large tabular datasets, ensuring both scalability and reproducibility. The data preparation protocol and the training set are available in our Zenodo repositories.

Model development pipeline and MLOps framework

All modeling tasks were conducted within the SAS Viya 4. environment, following a fully automated MLOps workflow (Figure S1). For each target, we employed tree-based algorithms—gradient boosting (GB) and random forest (RF)—alongside feed-forward neural networks (MLPs).. The dataset was split into 80/20 training and test partitions (dependent variable stratification and scaffold-based diversity sampling), with the test set held out entirely from model training and hyperparameter optimization to ensure unbiased external validation.

Hyperparameter tuning was performed using the SAS Autotune procedure, which combines Latin Hypercube Sampling and a genetic algorithm to efficiently explore the parameter space. Tuning was executed exclusively on the training set using fivefold cross-validation, with RMSE as the optimization objective. Early stopping was triggered after 360 minutes or five stagnant iterations.

Model robustness was assessed through multiple internal validation strategies, including fivefold cross-validation, 90/10 bootstrap sampling, and y-randomization, and two train/test splits with dependent variable stratification and scaffold-based diversity sampling, each iterated 100 times. Performance metrics included Pearson correlation (r), determination coefficient (R^2), root mean squared error (RMSE), and enrichment factor (EF). A model was deployed if it met the following criteria: $r \geq 0.7$, $R^2 \geq 0.5$, and RMSE ≤ 20% of the target values range. The model with the highest Pearson correlation was selected as the champion model for the given target. Champion models were automatically published and versioned within the ProfhEX platform. The MLOps process was fully automated end-to-end, covering hyperparameter tuning, validation, champion model selection, and model promotion or updating. For further details, please refer to the ProfhEX earlier work.[1]

The applicability domain (AD) of each model was determined using a well-established, QSAR-standard approach based on the consensus of three complementary methods. These include the position of a query molecule within a PCA-defined bounding box of the training set physicochemical descriptors, a penalty for the presence of fragments not observed in the training data, and the average similarity of the query to its three closest training set analogues based on ECFP4 Tanimoto metrics. The consensus of these criteria produces a composite score that reflects the reliability of predictions for each compound. Molecules with a score above 0.75 were considered inside the AD.[1]

ProfhEX web interface implementation

The protocol described above is freely accessible at https://profhex.exscalate.eu/. The web interface, built on the open-source LAMP stack (Linux, Apache, MariaDB, PHP), provides a seamless and responsive user experience for viewing and interacting with output data, which are dynamically generated in a fully automated Pipeline Pilot and SAS Viya 4 workflow.

Supporting figures and tables

Figure S1. ProfhEX workflow for model development, validation, and deployment. Created in BioRender.

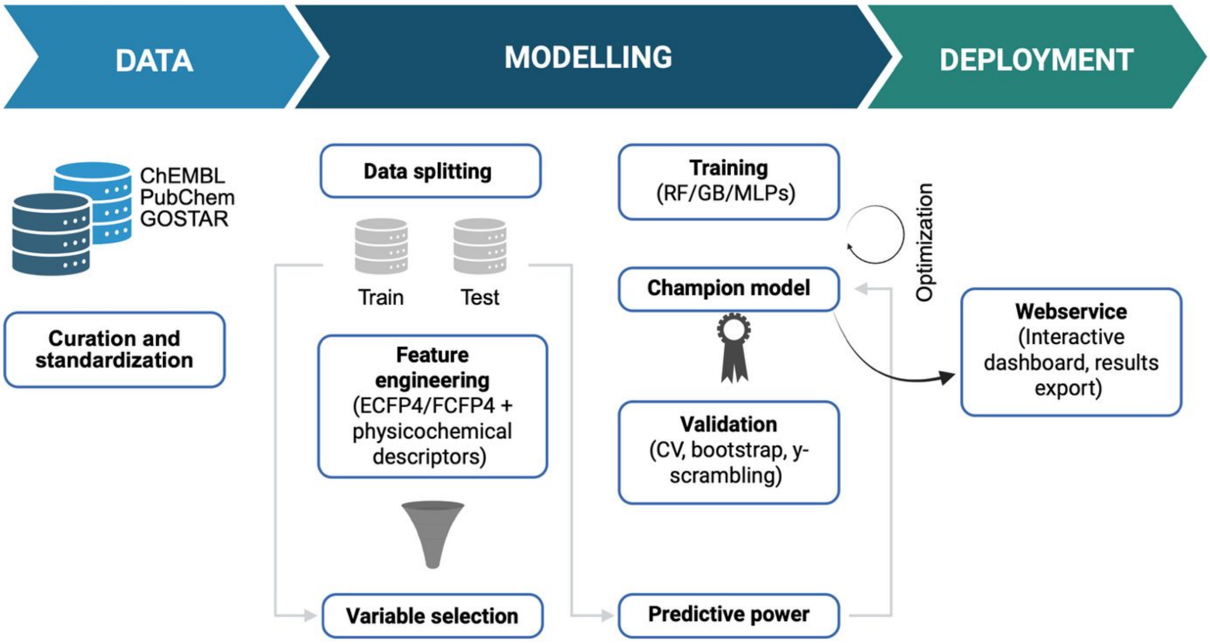

Table S1. Overview of ProfhEX models grouped by protein family. The table reports the average dataset size (Avg(N)), determination coefficient (Avg(R^2)), and root mean squared error (Avg(RMSE)) for each protein family.

l1	l2	Count	Avg(N)	Avg(R2)	Avg(RMSE)
Adhesion	Oth	4	272	0.67	0.82
Aux Transp	Fatty Acid Binding	3	437	0.74	0.54
Cytosolic Prot	Oth	18	979	0.68	0.66
Enzyme	Ligase	1	7076	0.71	0.5
Enzyme	Phosphatase	8	1257	0.72	0.6
Enzyme	Oth	36	1021	0.69	0.6
Enzyme	Aminoacyltransferase	2	1002	0.79	0.62
Enzyme	Kinase	165	1930	0.68	0.64
Enzyme	Transferase	42	1588	0.67	0.65
Enzyme	Phosphodiesterase	17	1849	0.69	0.66
Enzyme	Oxidoreductase	36	1542	0.68	0.69
Enzyme	Protease	108	1872	0.69	0.73
Enzyme	Hydrolase	23	1294	0.66	0.73
Enzyme	Isomerase	7	840	0.67	0.76
Enzyme	CYPs	12	1977	0.59	0.78
Enzyme	Lyase	16	2283	0.64	0.83
Epigen Reg	Writer	8	891	0.65	0.57
Epigen Reg	Reader	10	753	0.72	0.58
Epigen Reg	Eraser	13	2309	0.65	0.63
Ion Channel	Ligand Gated	12	2058	0.63	0.6
Ion Channel	Voltage Gated	27	2330	0.66	0.61
Ion Channel	Oth	7	1380	0.79	0.62
Membrane rcpt	GPCR Frizzled	2	910	0.62	0.48
Membrane rcpt	Toll-like and Il-1	5	5872	0.79	0.52
Membrane rcpt	GPCR family B	8	2026	0.78	0.61
Membrane rcpt	Oth	7	1086	0.68	0.61
Membrane rcpt	GPCR family A	236	2046	0.68	0.69
Membrane rcpt	GPCR family C	11	1931	0.64	0.7
Nuclear Prot	Oth	3	1463	0.76	0.79
Secreted Prot	Oth	6	891	0.74	0.56
Transc Factor	Oth	6	817	0.66	0.57
Transc Factor	Nuclear rcpt	36	2374	0.7	0.72
Transp	Electrochem	22	1461	0.67	0.62
Transp	Primary active Transp	4	782	0.68	0.78

Figure S2. Model performance metrics by protein family. Bar chart displaying R^2 (blue) and RMSE (red) values for protein families displayed in Table S1.

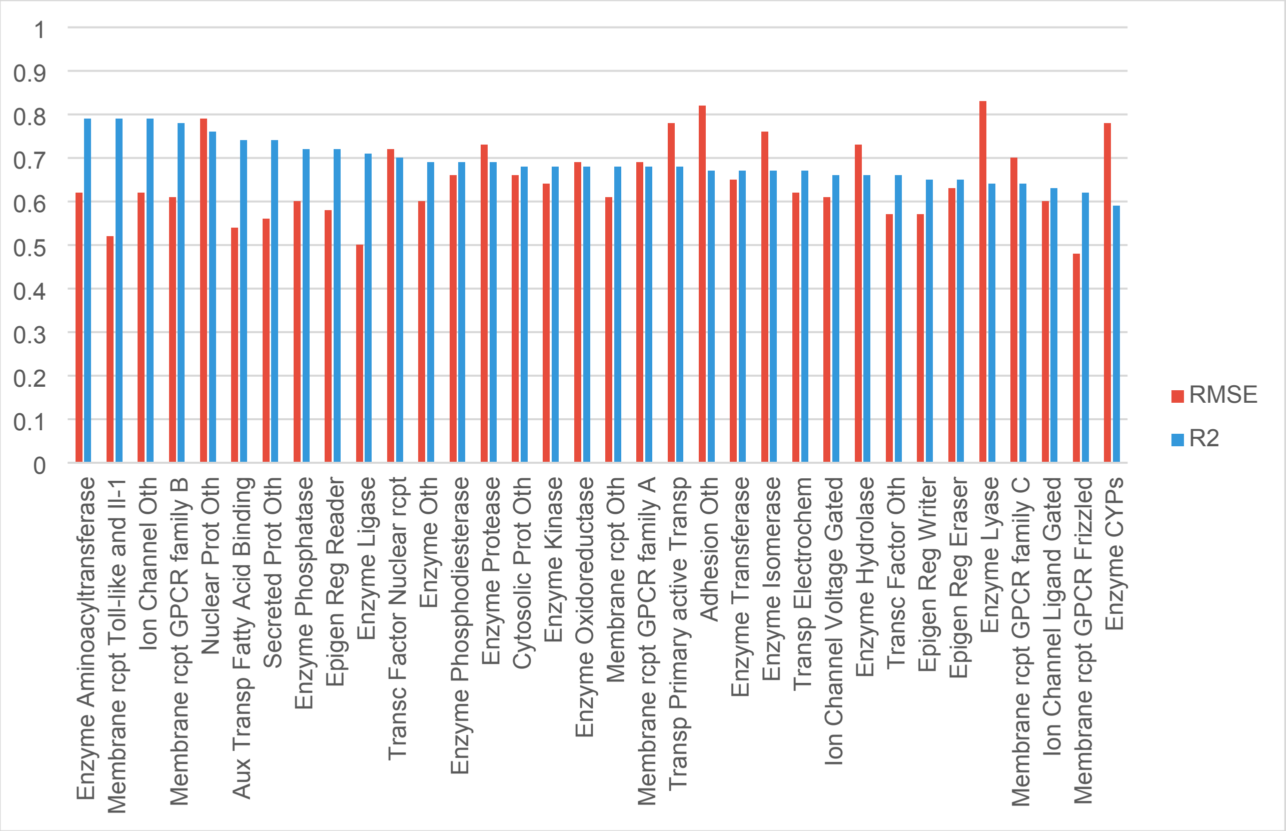

Table S2. Comprehensive list of ProfhEX models ordered by gene name. The table includes the training size and cross-validation performance metrics (EF, R^2, RMSE, r, MAE).

Gene	UniProt	Target Property	Training size	EF	R2	RMSE	r	MAE
**AADAT**	Q8N5Z0	PIC50	158	19.7504	0.4339	0.5023	0.6696	0.3692
**AAK1**	Q2M2I8	PIC50	329	11.7498	0.5596	0.605	0.7507	0.4496
**ABCB1**	P08183	PEC50	286	6.8095	0.6146	0.967	0.794	0.6239
**ABCB1**	P08183	PIC50	386	5.079	0.6373	0.7181	0.8	0.4942
**ABCB1**	P08183	PKI	96	19.2003	0.4821	0.6609	0.7137	0.482
**ABCC1**	P33527	PIC50	107	21.3995	0.5856	0.6742	0.7756	0.4823
**ABCG2**	Q9UNQ0	PEC50	158	6.5835	0.8626	0.7536	0.9313	0.5556
**ABCG2**	Q9UNQ0	PIC50	277	13.1903	0.4727	0.6254	0.6946	0.4491
**ABL1**	P00519	PKI	314	13.0835	0.6876	0.847	0.8328	0.6352
**ABL1**	P00519	PIC50	543	9.1413	0.7728	0.7363	0.8799	0.5328
**ACACA**	Q13085	PIC50	298	19.8669	0.6332	0.5638	0.7991	0.4137
**ACACB**	O00763	PIC50	2122	15.7288	0.7052	0.5024	0.8403	0.3855
**ACE**	P12821	PIC50	235	11.7498	0.7363	1.1442	0.8611	0.8296
**ACE2**	Q9BYF1	PKI	42	0	0.5125	1.1533	0.7207	0.7414
**ACHE**	P22303	PIC50	1844	13.5429	0.6581	0.9319	0.8143	0.684
**ACHE**	P22303	PKI	227	16.5094	0.6527	1.1459	0.8122	0.8269
**ACKR3**	P25106	PEC50	158	13.1669	0.4309	0.6183	0.6672	0.4854
**ACKR3**	P25106	PIC50	206	10.3003	0.5358	0.4947	0.7369	0.3463
**ACR**	P10323	PIC50	66	21.9993	0.842	0.6757	0.9238	0.5122
**ACVRL1**	P37023	PIC50	80	0	0.5604	0.6039	0.7664	0.4363
**ADAM17**	P78536	PKI	453	8.7534	0.5826	0.6512	0.7685	0.4677
**ADAM17**	P78536	PIC50	480	14.0002	0.6971	0.6443	0.8366	0.4304
**ADAMTS4**	O75173	PIC50	267	12.3228	0.6648	0.5947	0.8185	0.4191
**ADAMTS5**	Q9UNA0	PIC50	469	15.8597	0.7156	0.4765	0.8474	0.3321
**ADAMTS7**	Q9UKP4	PIC50	75	9.3749	0.5308	0.4538	0.7519	0.3524
**ADH5**	P11766	PIC50	69	0	0.4778	0.2603	0.7549	0.1621
**ADORA1**	P30542	PEC50	111	0	0.6279	0.9487	0.802	0.6782

**ADORA1**	P30542	PKI	2439	11.016	0.562	0.7049	0.7503	0.5201
**ADORA2A**	P29274	PKI	3567	12.1363	0.7184	0.6828	0.8482	0.4962
**ADORA2A**	P29274	PIC50	680	1.4286	0.6213	0.599	0.7898	0.4178
**ADORA2B**	P29275	PIC50	696	9.9428	0.5693	0.564	0.756	0.3872
**ADORA2B**	P29275	PKI	995	11.9397	0.6156	0.6045	0.788	0.4115
**ADORA3**	P0DMS8	PKI	1988	8.5342	0.6587	0.7382	0.8128	0.5379
**ADRA1A**	P35348	PIC50	108	10.7997	0.5803	0.7965	0.7704	0.5821
**ADRA1A**	P35348	PKI	652	7.5989	0.6182	0.7149	0.7872	0.4848
**ADRA1B**	P35368	PKI	580	11.6665	0.6701	0.6706	0.8206	0.4807
**ADRA2A**	P08913	PIC50	505	18.18	0.7314	0.551	0.8564	0.3756
**ADRA2A**	P08913	PEC50	82	10.2501	0.4097	1.0917	0.685	0.8257
**ADRA2B**	P18089	PIC50	161	20.1245	0.6736	0.7262	0.8259	0.5163
**ADRA2B**	P18089	PEC50	101	10.1	0.7057	0.6496	0.8466	0.4825
**ADRA2B**	P18089	PKI	334	16.8404	0.5755	0.7733	0.7624	0.5482
**ADRA2C**	P18825	PEC50	104	10.3999	0.5938	0.8577	0.7791	0.6252
**ADRA2C**	P18825	PIC50	538	14.4917	0.7026	0.679	0.8402	0.4756
**ADRA2C**	P18825	PKI	348	11.6975	0.5533	0.7908	0.7469	0.5675
**ADRB1**	P08588	PIC50	243	20.2501	0.6734	0.6215	0.8248	0.4293
**ADRB1**	P08588	PKI	175	9.7223	0.6494	0.8044	0.8104	0.5599
**ADRB2**	P07550	PIC50	301	6.6887	0.8447	0.5951	0.9198	0.3818
**ADRB2**	P07550	PEC50	646	12.423	0.7254	0.6909	0.8529	0.4742
**ADRB2**	P07550	PKI	229	12.4909	0.612	0.8279	0.7854	0.5302
**ADRB3**	P13945	PEC50	1034	14.2036	0.641	0.7895	0.8022	0.5822
**ADRB3**	P13945	PKI	123	10.2501	0.4476	0.825	0.6835	0.6304
**AGTR1**	P30556	PIC50	467	15.7922	0.8047	0.7388	0.8979	0.4883
**AGTR2**	P50052	PIC50	127	14.1111	0.4623	0.7396	0.7142	0.5553
**AHR**	P35869	PEC50	89	11.125	0.4529	0.8615	0.7033	0.6673
**AHR**	P35869	PIC50	461	13.3622	0.593	0.6517	0.7727	0.4887
**AKR1A1**	P14550	PIC50	142	6.7618	0.5256	0.7529	0.7355	0.5787
**AKR1B1**	P15121	PIC50	338	17.0421	0.6103	0.8181	0.7841	0.6187
**AKR1C3**	P42330	PIC50	369	8.7858	0.7141	0.6685	0.8477	0.4834
**AKT1**	P31749	PIC50	1047	9.5881	0.6699	0.7186	0.8194	0.5431
**AKT1**	P31749	PKI	92	9.2001	0.5558	0.9478	0.762	0.6733

**AKT2**	P31751	PIC50	325	17.4111	0.667	0.6268	0.8187	0.4675
**AKT3**	Q9Y243	PIC50	87	0	0.5134	0.8598	0.7339	0.5989
**ALDH2**	P05091	PIC50	93	0	0.6098	0.7934	0.8002	0.6122
**ALK**	Q9UM73	PKI	144	6.8573	0.7047	0.8349	0.8442	0.6111
**ALK**	Q9UM73	PIC50	1099	12.7158	0.7498	0.5713	0.8674	0.3741
**ALOX15**	P16050	PIC50	159	6.625	0.5622	0.6989	0.7572	0.5265
**ALOX5**	P09917	PIC50	1146	9.6155	0.5898	0.7035	0.7693	0.4928
**ALOX5AP**	P20292	PIC50	607	10.1169	0.6621	0.5355	0.8149	0.3664
**ALOX5AP**	P20292	PKI	421	10.024	0.586	0.5963	0.7682	0.4546
**ALPL**	P05186	PIC50	183	10.1666	0.8622	0.4833	0.9308	0.3266
**ANO1**	Q5XXA6	PEC50	97	19.3996	0.5224	0.3922	0.7401	0.3035
**ANPEP**	P15144	PIC50	110	18.3332	0.5707	0.7347	0.7666	0.5057
**AOC1**	P19801	PIC50	201	0	0.6915	0.6623	0.8348	0.4945
**AOC3**	Q16853	PIC50	625	7.7543	0.7771	0.4889	0.8823	0.3164
**APLNR**	P35414	PEC50	1310	2.2902	0.7813	0.8774	0.8844	0.6071
**APP**	P05067	PIC50	863	12.9861	0.7792	0.5484	0.8834	0.3958
**APP**	P05067	PEC50	284	20.2855	0.8263	0.4384	0.9115	0.2873
**APP**	P05067	PKI	198	4.95	0.6189	0.7257	0.7913	0.5401
**AR**	P10275	PKI	441	13.3637	0.6044	0.6324	0.7793	0.4394
**AR**	P10275	PIC50	1498	13.3154	0.6771	0.6178	0.8238	0.4444
**ASAH1**	Q13510	PIC50	54	17.9995	0.4887	0.6493	0.7396	0.4604
**ATAD2**	Q6PL18	PIC50	49	24.5002	0.5595	0.6255	0.7738	0.4702
**ATM**	Q13315	PIC50	142	6.7618	0.6573	0.7896	0.816	0.5525
**ATR**	Q13535	PIC50	304	16.8888	0.5466	0.6065	0.7439	0.4345
**AURKA**	O14965	PKI	217	0	0.5611	0.7797	0.7545	0.5795
**AURKA**	O14965	PIC50	1001	9.009	0.6853	0.7705	0.8286	0.561
**AURKB**	Q96GD4	PIC50	691	9.8717	0.6896	0.7044	0.8316	0.5299
**AVPR1A**	P37288	PIC50	862	8.2543	0.6627	0.609	0.815	0.402
**AVPR1A**	P37288	PKI	825	8.2855	0.6191	0.6622	0.7885	0.4733
**AVPR1A**	P37288	PEC50	47	23.4996	0.4806	0.8742	0.7184	0.6431
**AVPR1B**	P47901	PKI	237	15.8	0.7546	0.5042	0.8707	0.3551
**AVPR1B**	P47901	PIC50	264	16.2465	0.6364	0.7878	0.8035	0.5734
**AVPR2**	P30518	PEC50	106	21.1999	0.6327	0.8723	0.8037	0.5996

**AVPR2**	P30518	PIC50	623	10.0482	0.7309	0.5509	0.8561	0.3853
**AVPR2**	P30518	PKI	164	0	0.668	0.8008	0.8206	0.4468
**AXL**	P30530	PIC50	453	15.3185	0.7754	0.4606	0.8816	0.2905
**AXL**	P30530	PKI	174	6.4446	0.6895	0.7639	0.8376	0.5647
**BACE1**	P56817	PIC50	4723	14.9028	0.8745	0.6815	0.9355	0.4567
**BACE1**	P56817	PKI	728	5.3927	0.6347	0.7628	0.8046	0.5614
**BACE1**	P56817	PEC50	115	19.167	0.6555	0.5397	0.8182	0.4139
**BACE2**	Q9Y5Z0	PIC50	779	16.2293	0.7165	0.662	0.8476	0.4887
**BACE2**	Q9Y5Z0	PKI	450	15.2174	0.7159	0.621	0.8474	0.4536
**BCAT2**	O15382	PIC50	44	0	0.6029	0.6138	0.7928	0.429
**BCHE**	P06276	PIC50	1107	11.8931	0.5698	1.011	0.7624	0.6783
**BCHE**	P06276	PKI	138	19.7144	0.6173	0.9346	0.7919	0.6274
**BCL2**	P10415	PIC50	468	9.0434	0.8687	0.5471	0.9329	0.3998
**BCL2**	P10415	PKI	368	11.6824	0.9143	0.6397	0.9566	0.4598
**BCL2L1**	Q07817	PIC50	309	20.6005	0.8344	0.6405	0.9148	0.4737
**BCL2L1**	Q07817	PKI	278	6.619	0.8791	0.8044	0.9386	0.5594
**BCL6**	P41182	PIC50	211	14.3866	0.8473	0.396	0.9227	0.2911
**BDKRB1**	P46663	PKI	408	15.2999	0.7396	0.5953	0.8612	0.3928
**BDKRB1**	P46663	PIC50	812	13.6162	0.7617	0.597	0.8734	0.4367
**BDKRB2**	P30411	PKI	299	16.611	0.8628	0.5684	0.9297	0.3371
**BIRC2**	Q13490	PIC50	165	6.8749	0.7373	0.6259	0.8615	0.4366
**BIRC3**	Q13489	PIC50	67	0	0.8331	0.4425	0.9187	0.2983
**BMP1**	P13497	PIC50	204	5.1	0.6415	0.9286	0.8074	0.6424
**BMX**	P51813	PIC50	68	0	0.5749	0.9081	0.7768	0.6087
**BRAF**	P15056	PEC50	74	18.4993	0.4261	0.6633	0.6819	0.4708
**BRAF**	P15056	PIC50	1452	10.9743	0.6644	0.7012	0.8157	0.4926
**BRD2**	P25440	PIC50	139	6.619	0.6857	0.5671	0.8343	0.3771
**BRD3**	Q15059	PIC50	126	0	0.6632	0.5041	0.8218	0.32
**BRD4**	O60885	PKI	332	5.5799	0.6925	0.6654	0.835	0.5022
**BRD4**	O60885	PIC50	1161	7.8326	0.8148	0.4873	0.9035	0.3215
**BRPF1**	P55201	PIC50	66	21.9993	0.5658	0.5724	0.7709	0.4275
**BRS3**	P32247	PEC50	104	0	0.5585	0.7053	0.7592	0.5267
**BRS3**	P32247	PIC50	47	0	0.5478	0.6404	0.7724	0.5138

**BTK**	Q06187	PIC50	2096	13.7833	0.6303	0.6644	0.7948	0.4663
**BTK**	Q06187	PKI	92	18.4001	0.7139	0.5872	0.8504	0.3175
**BUB1**	O43683	PIC50	685	7.1954	0.74	0.5784	0.8614	0.4237
**C1S**	P09871	PIC50	55	18.3332	0.7592	0.4768	0.8784	0.2387
**C1S**	P09871	PKI	36	17.9999	0.7863	0.6457	0.9056	0.4469
**C5**	P01031	PKI	117	9.75	0.816	0.6124	0.9082	0.4877
**CA1**	P00915	PIC50	165	20.6248	0.694	0.7469	0.8377	0.5309
**CA1**	P00915	PKI	2388	14.632	0.5434	0.8317	0.7376	0.5888
**CA12**	O43570	PKI	1369	14.9132	0.597	0.7272	0.7734	0.5421
**CA13**	Q8N1Q1	PKI	122	20.3339	0.6421	0.946	0.8085	0.6676
**CA14**	Q9ULX7	PKI	237	11.85	0.747	0.857	0.8668	0.6215
**CA2**	P00918	PIC50	549	12.9397	0.6976	0.7329	0.8377	0.5029
**CA2**	P00918	PKI	2702	17.0498	0.6583	0.8254	0.812	0.5798
**CA3**	P07451	PKI	91	9.1	0.4882	1.0953	0.714	0.7426
**CA4**	P22748	PKI	427	15.815	0.7691	0.7911	0.8784	0.5861
**CA4**	P22748	PIC50	169	7.0419	0.581	0.9441	0.7693	0.6574
**CA5A**	P35218	PKI	198	9.9	0.5358	1.1193	0.74	0.7558
**CA5B**	Q9Y2D0	PKI	165	20.6248	0.7579	0.9212	0.8737	0.6622
**CA7**	P43166	PKI	446	20.2731	0.6959	0.8711	0.8364	0.6338
**CA9**	Q16790	PKI	1740	16	0.639	0.7591	0.8002	0.5615
**CACNA1B**	Q00975	PIC50	908	19.0566	0.6829	0.405	0.8273	0.2931
**CACNA1G**	O43497	PIC50	548	18.4512	0.7509	0.5617	0.8678	0.4055
**CACNA1H**	O95180	PIC50	715	14.1868	0.7823	0.4969	0.8858	0.3542
**CACNA1I**	Q9P0X4	PIC50	132	12.5716	0.8594	0.5555	0.9287	0.3463
**CALCRL**	Q16602	PIC50	613	14.8305	0.8481	0.7256	0.9214	0.4991
**CALCRL**	Q16602	PKI	399	14.9626	0.8438	0.7013	0.9197	0.4821
**CAPN1**	P07384	PIC50	308	6.8443	0.5923	0.6509	0.773	0.4293
**CAPN1**	P07384	PKI	128	14.2219	0.6309	0.6727	0.8009	0.4454
**CARM1**	Q86X55	PIC50	276	6.5715	0.7743	0.3817	0.8818	0.2181
**CASP1**	P29466	PIC50	191	9.5502	0.7368	0.8323	0.8615	0.5956
**CASP1**	P29466	PKI	100	10	0.5356	0.9794	0.7485	0.7263
**CASP3**	P42574	PIC50	600	20	0.7711	0.755	0.8795	0.5122
**CASP6**	P55212	PIC50	52	17.3331	0.6092	0.7143	0.8066	0.5212

**CASP7**	P55210	PIC50	156	19.4998	0.6746	0.8304	0.8263	0.5591
**CASP8**	Q14790	PIC50	97	0	0.6341	0.7149	0.8086	0.4815
**CASR**	P41180	PIC50	427	20.3336	0.5942	0.8436	0.7733	0.552
**CASR**	P41180	PEC50	230	7.6667	0.5314	0.6713	0.7439	0.4633
**CCKAR**	P32238	PIC50	157	19.6253	0.609	0.8808	0.7914	0.5591
**CCKAR**	P32238	PEC50	72	0	0.7243	0.6299	0.8584	0.335
**CCKBR**	P32239	PKI	201	5.0251	0.4876	0.9129	0.705	0.6507
**CCKBR**	P32239	PIC50	282	10.0712	0.6554	0.924	0.8137	0.6688
**CCR1**	P32246	PIC50	546	11.03	0.5811	0.7238	0.7641	0.5236
**CCR2**	P41597	PKI	262	12.0923	0.6466	0.6321	0.8072	0.4623
**CCR2**	P41597	PIC50	1599	12.4919	0.6955	0.7125	0.8346	0.4922
**CCR2**	P41597	PEC50	116	9.6669	0.4413	0.4469	0.7433	0.2315
**CCR3**	P51677	PKI	223	15.2047	0.6805	0.5873	0.8291	0.428
**CCR3**	P51677	PIC50	629	14.047	0.7297	0.7931	0.8557	0.5581
**CCR4**	P51679	PIC50	252	11.6309	0.5329	0.9775	0.7599	0.6777
**CCR5**	P51681	PIC50	1478	12.6494	0.8405	0.7123	0.9172	0.4623
**CCR6**	P51684	PIC50	315	9.8436	0.8357	0.4532	0.9158	0.3272
**CCR9**	P51686	PKI	79	0	0.4885	0.5094	0.7245	0.3891
**CD274**	Q9NZQ7	PIC50	215	0	0.7499	0.4123	0.867	0.1855
**CD38**	P28907	PIC50	90	18.0002	0.8339	0.4319	0.916	0.2789
**CDC25B**	P30305	PIC50	191	0	0.4176	0.5647	0.6633	0.4126
**CDC7**	O00311	PKI	114	9.4999	0.4528	0.4564	0.6817	0.2563
**CDK1**	P06493	PIC50	392	12.2501	0.626	0.867	0.7934	0.6458
**CDK2**	P24941	PIC50	596	4.9667	0.6866	0.7765	0.8299	0.566
**CDK4**	P11802	PIC50	282	3.3571	0.7288	0.7308	0.8562	0.5224
**CDK6**	Q00534	PIC50	113	0	0.6811	0.7505	0.8326	0.5337
**CDK9**	P50750	PIC50	283	10.1073	0.6023	0.6381	0.7805	0.4398
**CES1**	P23141	PIC50	52	17.3331	0.4348	1.0136	0.7073	0.7857
**CETP**	P11597	PIC50	951	12.5131	0.8284	0.6706	0.9107	0.461
**CFB**	P00751	PIC50	153	12.7499	0.4151	0.7696	0.6635	0.6063
**CFD**	P00746	PIC50	620	5	0.5565	0.5912	0.7506	0.4323
**CFTR**	P13569	PEC50	424	10.0952	0.6714	0.6579	0.8215	0.4783
**CGAS**	Q8N884	PIC50	341	2.8655	0.7029	0.4814	0.8415	0.3614

**CHEK1**	O14757	PKI	223	10.1365	0.8376	0.9122	0.9171	0.7097
**CHEK1**	O14757	PIC50	668	15.5712	0.466	1.2074	0.6884	0.8072
**CHEK2**	O96017	PIC50	167	6.9584	0.6854	0.6282	0.8335	0.4585
**CHIA**	Q9BZP6	PIC50	81	20.2503	0.6229	0.6305	0.801	0.4559
**CHIT1**	Q13231	PIC50	49	0	0.5394	0.6532	0.7498	0.4024
**CHKA**	P35790	PIC50	165	20.6248	0.8138	0.4735	0.9062	0.3489
**CHRM1**	P11229	PKI	820	6.2501	0.6577	1.1131	0.8133	0.6524
**CHRM1**	P11229	PIC50	321	3.3437	0.6928	0.8129	0.8357	0.5011
**CHRM1**	P11229	PEC50	1002	11.022	0.6566	0.704	0.8113	0.4584
**CHRM2**	P08172	PEC50	363	11.5237	0.6107	0.5773	0.7845	0.3888
**CHRM2**	P08172	PIC50	286	3.4048	0.773	0.8746	0.8814	0.5254
**CHRM2**	P08172	PKI	1081	11.8289	0.6362	0.8941	0.7987	0.5199
**CHRM3**	P20309	PIC50	737	3.9837	0.807	0.6773	0.8987	0.4387
**CHRM3**	P20309	PEC50	113	9.4167	0.8597	0.6854	0.9287	0.3473
**CHRM3**	P20309	PKI	1083	5.4697	0.7584	0.8326	0.8723	0.4795
**CHRM4**	P08173	PEC50	879	9.9885	0.5437	0.5846	0.7405	0.4449
**CHRM4**	P08173	PIC50	340	14.2859	0.5529	0.7874	0.7478	0.5766
**CHRNA7**	P36544	PEC50	992	18.8483	0.651	0.6347	0.8075	0.4245
**CHRNA7**	P36544	PKI	295	9.8333	0.487	1.0023	0.7038	0.7108
**CLK1**	P49759	PIC50	154	6.4165	0.6153	0.4204	0.7876	0.2586
**CLK4**	Q9HAZ1	PIC50	66	0	0.7263	0.5923	0.8586	0.3929
**CMA1**	P23946	PIC50	399	14.9626	0.6394	0.7045	0.8026	0.5077
**CNR1**	P21554	PIC50	1728	10.9074	0.7967	0.743	0.8931	0.4913
**CNR1**	P21554	PKI	3014	12.3089	0.7272	0.8746	0.8533	0.5938
**CNR1**	P21554	PEC50	1668	10.3528	0.6663	0.7574	0.8168	0.5399
**CNR2**	P34972	PKI	2602	13.0867	0.751	0.7389	0.8672	0.5182
**CNR2**	P34972	PIC50	1008	10.0801	0.7337	0.657	0.8571	0.4359
**CNR2**	P34972	PEC50	2217	6.3549	0.6821	0.7333	0.8265	0.5317
**COMT**	P21964	PIC50	173	12.8148	0.459	0.5552	0.6867	0.3864
**CPB2**	Q96IY4	PIC50	84	10.4998	0.4516	1.2327	0.7023	0.9223
**CREBBP**	Q92793	PIC50	150	18.75	0.953	0.4551	0.9765	0.2368
**CRHR1**	P34998	PIC50	1125	12.2285	0.7995	0.5807	0.8944	0.315
**CRHR1**	P34998	PKI	496	9.9201	0.6012	0.6688	0.7773	0.4848

**CSF1R**	P07333	PKI	138	6.5715	0.5806	0.7885	0.7708	0.5704
**CSF1R**	P07333	PIC50	684	15.8066	0.7287	0.483	0.8543	0.3083
**CSNK1A1**	P48729	PIC50	116	9.6669	0.7386	0.4671	0.8682	0.3188
**CSNK1D**	P48730	PIC50	423	15.1068	0.7547	0.5638	0.8704	0.4106
**CSNK1E**	P49674	PIC50	180	15.0002	0.6759	0.5812	0.8265	0.4148
**CSNK2A1**	P68400	PIC50	335	8.4452	0.6332	0.746	0.7988	0.5596
**CSNK2A1**	P68400	PKI	119	19.833	0.4095	0.9692	0.6652	0.7274
**CSNK2A2**	P19784	PIC50	48	0	0.6125	0.9057	0.807	0.6872
**CTRC**	Q99895	PIC50	67	0	0.4799	0.9575	0.7265	0.6957
**CTRC**	Q99895	PKI	46	23.0001	0.4443	0.9341	0.7152	0.7143
**CTSA**	P10619	PIC50	484	14.1168	0.5934	0.5656	0.7727	0.4223
**CTSB**	P07858	PIC50	437	8.8283	0.6181	0.8081	0.788	0.5597
**CTSB**	P07858	PKI	245	4.0834	0.5538	1.1368	0.762	0.8483
**CTSC**	P53634	PIC50	511	17.6889	0.6955	0.6379	0.8356	0.4618
**CTSD**	P07339	PIC50	829	17.8404	0.8274	0.7672	0.9102	0.4891
**CTSD**	P07339	PKI	1382	19.3141	0.5915	0.3394	0.7828	0.1066
**CTSG**	P08311	PIC50	114	18.9999	0.6278	0.8112	0.8009	0.5545
**CTSK**	P43235	PIC50	767	13.4559	0.7375	0.6289	0.8594	0.3889
**CTSK**	P43235	PKI	378	14.9211	0.6972	0.6508	0.8369	0.4102
**CTSL**	P07711	PIC50	734	10.5802	0.736	0.7439	0.8589	0.509
**CTSL**	P07711	PKI	356	14.1269	0.5548	0.9359	0.7513	0.6914
**CTSS**	P25774	PIC50	1602	14.393	0.7432	0.5925	0.8627	0.408
**CTSS**	P25774	PKI	1092	16.2442	0.7403	0.5741	0.8617	0.3351
**CXCR3**	P49682	PKI	114	9.4999	0.8478	0.5696	0.9225	0.4056
**CXCR3**	P49682	PIC50	706	1.4408	0.6731	0.5312	0.8214	0.3714
**CXCR4**	P61073	PIC50	572	14.3447	0.6628	0.7005	0.8159	0.4511
**CXCR5**	P32302	PIC50	73	18.2495	0.8773	0.6501	0.9397	0.3661
**CYP11B1**	P15538	PIC50	402	15.0754	0.5696	0.7903	0.757	0.5811
**CYP11B1**	P15538	PEC50	363	17.2855	0.7409	0.6712	0.8625	0.5154
**CYP11B2**	P19099	PIC50	732	11.8702	0.5411	1.0312	0.738	0.7213
**CYP11B2**	P19099	PEC50	381	12.5331	0.7107	0.7695	0.8466	0.5635
**CYP19A1**	P11511	PIC50	862	9.4335	0.5591	0.9605	0.7491	0.7059
**CYP1A1**	P04798	PIC50	116	19.3338	0.5704	0.7266	0.767	0.5572

**CYP1A2**	P05177	PIC50	535	5.404	0.5194	0.7397	0.7228	0.5454
**CYP1B1**	Q16678	PIC50	138	13.1429	0.5288	0.8844	0.7386	0.6608
**CYP3A4**	P08684	PIC50	2072	12.6339	0.6745	0.6306	0.8217	0.4399
**CYP4F2**	P78329	PIC50	209	5.2249	0.5488	0.6698	0.7478	0.528
**CYP8B1**	Q9UNU6	PIC50	63	0	0.5128	0.8372	0.7422	0.6184
**CYSLTR1**	Q9Y271	PIC50	79	9.8752	0.5648	0.7899	0.7676	0.5659
**DAGLA**	Q9Y4D2	PIC50	61	0	0.5402	0.8266	0.7566	0.6295
**DGAT1**	O75907	PIC50	1386	14.3478	0.7543	0.7297	0.8698	0.5117
**DGAT2**	Q96PD7	PIC50	457	11.0385	0.5377	0.637	0.7358	0.5031
**DGKA**	P23743	PIC50	691	16.9229	0.717	0.6654	0.8477	0.5065
**DHFR**	P00374	PKI	243	16.2001	0.7521	0.9575	0.8691	0.6567
**DHODH**	Q02127	PKI	60	0	0.7435	0.7718	0.876	0.6132
**DHODH**	Q02127	PIC50	693	9.9	0.7402	0.713	0.8616	0.5254
**DPP4**	P27487	PKI	654	9.1467	0.6971	0.7962	0.836	0.4701
**DPP4**	P27487	PIC50	1859	9.7246	0.6968	0.7489	0.8352	0.5268
**DPP7**	Q9UHL4	PIC50	381	20.053	0.7034	0.6674	0.8411	0.4744
**DPP8**	Q6V1X1	PKI	43	0	0.7894	0.7156	0.9017	0.5553
**DPP8**	Q6V1X1	PIC50	341	17.1932	0.5897	0.6498	0.772	0.477
**DPP9**	Q86TI2	PIC50	243	16.2001	0.5815	0.6871	0.7668	0.4776
**DRD2**	P14416	PKI	3157	6.9774	0.6323	0.7048	0.7972	0.5018
**DRD2**	P14416	PIC50	610	14.7582	0.4692	0.9342	0.6877	0.5799
**DRD3**	P35462	PKI	3464	13.0587	0.7273	0.5337	0.8539	0.3381
**DRD3**	P35462	PIC50	280	9.9998	0.5107	0.8791	0.7152	0.5918
**DRD4**	P21917	PKI	1080	16.3639	0.5208	0.8097	0.723	0.5976
**DRD4**	P21917	PIC50	261	0	0.6325	0.6468	0.797	0.4394
**DUT**	P33316	PIC50	74	18.4993	0.6113	0.4455	0.7972	0.3481
**DYRK1A**	Q13627	PEC50	201	15.0754	0.4963	0.4286	0.713	0.3339
**DYRK1A**	Q13627	PIC50	460	4.4445	0.5892	0.6346	0.7695	0.4609
**ECE1**	P42892	PIC50	166	13.8336	0.8168	0.7939	0.907	0.5048
**EDNRA**	P25101	PIC50	946	11.6526	0.7506	0.8447	0.8678	0.5975
**EDNRA**	P25101	PKI	107	0	0.572	1.0091	0.7656	0.757
**EDNRB**	P24530	PIC50	796	18.6558	0.7934	0.673	0.8914	0.4824
**EDNRB**	P24530	PKI	73	0	0.7176	0.7124	0.8559	0.5337

**EGFR**	P00533	PKI	178	9.8888	0.611	0.8468	0.7888	0.6264
**EGFR**	P00533	PIC50	3544	12.6903	0.7743	0.7674	0.8805	0.5439
**EGLN1**	Q9GZT9	PIC50	729	14.8501	0.8215	0.5211	0.907	0.3577
**EGLN2**	Q96KS0	PIC50	99	19.8	0.795	0.5218	0.8948	0.3212
**EGLN3**	Q9H6Z9	PIC50	206	20.6005	0.7344	0.5229	0.8595	0.3069
**EHMT1**	Q9H9B1	PIC50	52	0	0.4201	0.7298	0.6812	0.541
**EHMT2**	Q96KQ7	PIC50	199	4.9749	0.4705	0.5822	0.6934	0.3247
**EIF2AK3**	Q9NZJ5	PIC50	54	0	0.6371	0.6685	0.8086	0.4465
**ELANE**	P08246	PIC50	1081	9.0991	0.7923	0.6739	0.8905	0.4305
**ELANE**	P08246	PKI	669	12.4755	0.7321	0.9047	0.857	0.6656
**ENPP1**	P22413	PIC50	94	18.7996	0.7468	0.6151	0.8688	0.4074
**ENPP2**	Q13822	PIC50	1185	12.5531	0.6282	0.7773	0.7935	0.5093
**EP300**	Q09472	PIC50	76	18.9999	0.561	0.775	0.7668	0.579
**EPHA2**	P29317	PIC50	109	0	0.6601	0.4925	0.8189	0.33
**EPHX1**	P07099	PIC50	113	18.8335	0.542	0.8138	0.7434	0.6055
**EPHX2**	P34913	PIC50	931	16.681	0.7064	0.7491	0.8414	0.5424
**ERBB2**	P04626	PIC50	1484	10.6953	0.6856	0.6855	0.8286	0.4922
**ERBB4**	Q15303	PIC50	531	16.0906	0.736	0.5053	0.8597	0.2888
**ESR1**	P03372	PKI	227	4.1274	0.5874	0.8749	0.7731	0.6266
**ESR1**	P03372	PIC50	1502	9.346	0.7827	0.7855	0.8854	0.5564
**ESR1**	P03372	PEC50	540	0	0.8402	0.8124	0.9172	0.5162
**ESR2**	Q92731	PIC50	1137	11.2744	0.7779	0.8242	0.8827	0.5687
**ESR2**	Q92731	PKI	197	9.8498	0.5973	0.7884	0.7783	0.5952
**ESR2**	Q92731	PEC50	357	2.8333	0.8298	0.8311	0.9121	0.5146
**ESRRA**	P11474	PIC50	65	0	0.4476	0.6936	0.697	0.5067
**EZH2**	Q15910	PIC50	534	17.98	0.7782	0.698	0.8834	0.4934
**F10**	P00742	PIC50	1550	12.1795	0.7341	0.7249	0.8578	0.4959
**F10**	P00742	PKI	1755	11.9659	0.7594	0.8161	0.8725	0.5694
**F11**	P03951	PIC50	765	10.7368	0.7997	0.5545	0.895	0.3815
**F11**	P03951	PKI	720	8.5716	0.6417	0.7879	0.8023	0.5908
**F12**	P00748	PIC50	231	19.25	0.7125	0.731	0.8502	0.5632
**F2**	P00734	PIC50	1091	9.9182	0.644	0.8367	0.8034	0.5865
**F2**	P00734	PKI	1653	9.6559	0.7182	0.8811	0.8485	0.6056

**F2R**	P25116	PIC50	555	12.6135	0.6732	0.5973	0.8224	0.4339
**F2RL3**	Q96RI0	PIC50	246	8.2001	0.5616	0.8293	0.7565	0.6284
**F2RL3**	Q96RI0	PEC50	144	6.8573	0.5961	0.5742	0.7836	0.3926
**F7**	P08709	PIC50	271	11.6144	0.7543	0.6485	0.8744	0.473
**F7**	P08709	PKI	215	19.5451	0.8222	0.7279	0.9081	0.5328
**F9**	P00740	PIC50	79	0	0.6237	0.4984	0.7964	0.3025
**F9**	P00740	PKI	149	21.286	0.6437	0.8263	0.8121	0.6397
**FAAH**	O00519	PIC50	1087	10.0649	0.6958	0.8062	0.8363	0.6071
**FAAH**	O00519	PKI	252	19.3849	0.6124	0.5645	0.7865	0.3579
**FABP4**	P15090	PIC50	176	0	0.7236	0.5477	0.8545	0.4056
**FABP4**	P15090	PKI	85	10.6252	0.8305	0.607	0.917	0.3483
**FABP5**	Q01469	PIC50	131	6.238	0.6608	0.4605	0.8189	0.351
**FAP**	Q12884	PIC50	222	15.1364	0.736	0.6994	0.8615	0.5008
**FASN**	P49327	PIC50	1700	9.4118	0.7952	0.6102	0.8921	0.4121
**FBP1**	P09467	PIC50	182	10.1111	0.6221	0.661	0.7939	0.4873
**FFAR1**	O14842	PEC50	1385	10.036	0.6776	0.6263	0.8234	0.4341
**FFAR1**	O14842	PIC50	49	24.5002	0.6274	0.5241	0.8049	0.3117
**FFAR2**	O15552	PEC50	174	19.3338	0.5785	0.5985	0.771	0.4608
**FFAR4**	Q5NUL3	PEC50	792	13.6125	0.5118	0.4794	0.7176	0.3667
**FGFR1**	P11362	PIC50	1350	7.3529	0.7691	0.618	0.8774	0.4341
**FGFR2**	P21802	PIC50	771	10.5437	0.6727	0.475	0.8212	0.3149
**FGFR3**	P22607	PIC50	958	10.5044	0.7025	0.619	0.8398	0.4362
**FGFR4**	P22455	PIC50	525	7.3428	0.6225	0.5623	0.7905	0.4112
**FKBP1A**	P62942	PKI	130	12.3809	0.6276	0.9172	0.8041	0.7046
**FKBP1A**	P62942	PIC50	64	0	0.4371	0.8445	0.6989	0.6388
**FLT1**	P17948	PKI	108	10.7997	0.5784	0.9271	0.7743	0.7274
**FLT1**	P17948	PIC50	319	9.9686	0.6634	0.8041	0.8169	0.5716
**FLT3**	P36888	PIC50	1224	15.0491	0.8516	0.7397	0.9232	0.4405
**FNTA**	P49354	PIC50	180	10.0001	0.5715	0.9613	0.7644	0.7221
**FPR1**	P21462	PEC50	190	18.9997	0.7752	0.6746	0.8824	0.4465
**FPR1**	P21462	PKI	47	0	0.6036	0.6128	0.8094	0.456
**FPR2**	P25090	PEC50	835	16.3728	0.6942	0.673	0.8346	0.4969
**FPR3**	P25089	PEC50	121	0	0.5167	0.5104	0.7288	0.3921

**FSHR**	P23945	PEC50	384	2.5264	0.7452	0.6673	0.8651	0.4988
**FURIN**	P09958	PKI	129	14.3332	0.6782	0.5549	0.8279	0.3114
**GAA**	P10253	PIC50	110	0	0.6661	0.6692	0.8244	0.485
**GABRA5**	P31644	PKI	403	5.0375	0.6723	0.3629	0.8209	0.1906
**GCGR**	P47871	PIC50	926	6.3571	0.6976	0.6346	0.8364	0.4445
**GCGR**	P47871	PKI	495	0	0.9323	0.4087	0.9658	0.2005
**GCK**	P35557	PEC50	930	12.4973	0.7046	0.5566	0.8402	0.4048
**GGPS1**	O95749	PIC50	59	0	0.6834	0.693	0.8429	0.5099
**GHSR**	Q92847	PIC50	785	13.8383	0.4973	0.8396	0.7076	0.6356
**GHSR**	Q92847	PEC50	479	15.9665	0.6112	0.7938	0.7839	0.6038
**GLP1R**	P43220	PEC50	681	7.1534	0.7803	0.6352	0.8843	0.4175
**GLRA1**	P23415	PIC50	52	0	0.883	0.4723	0.9478	0.291
**GLS**	O94925	PIC50	295	13.1111	0.7322	0.3944	0.8576	0.2136
**GNRHR**	P30968	PIC50	862	12.971	0.754	0.7344	0.869	0.4998
**GNRHR**	P30968	PKI	331	5.563	0.5819	0.6603	0.7649	0.4625
**GPBAR1**	Q8TDU6	PEC50	1139	16.5074	0.5499	0.5068	0.7426	0.3173
**GPR119**	Q8TDV5	PEC50	2640	12.0753	0.746	0.6912	0.8642	0.4655
**GPR139**	Q6DWJ6	PKI	67	22.3339	0.5271	0.6073	0.7529	0.463
**GPR139**	Q6DWJ6	PEC50	123	0	0.8106	0.4004	0.903	0.2713
**GPR52**	Q9Y2T5	PEC50	98	9.8001	0.5311	0.4511	0.7386	0.3517
**GPR55**	Q9Y2T6	PEC50	75	18.7498	0.4777	0.7957	0.7132	0.6242
**GPR6**	P46095	PIC50	121	10.0833	0.88	0.6197	0.9403	0.4129
**GPR6**	P46095	PEC50	100	0	0.4459	0.5328	0.7044	0.4073
**GPR88**	Q9GZN0	PEC50	78	9.75	0.5702	0.5976	0.7725	0.4298
**GRB2**	P62993	PIC50	70	17.4997	0.6053	0.9595	0.7947	0.7307
**GRIN2B**	Q13224	PIC50	482	14.0583	0.5518	0.5443	0.7461	0.4006
**GRK6**	P43250	PIC50	134	0	0.7173	0.3936	0.8535	0.3049
**GRM1**	Q13255	PIC50	274	11.743	0.5142	0.7383	0.7315	0.5712
**GRM2**	Q14416	PIC50	693	14.1429	0.6921	0.5618	0.8329	0.3958
**GRM2**	Q14416	PKI	66	21.9993	0.6316	0.8612	0.8074	0.6209
**GRM2**	Q14416	PEC50	941	14.7527	0.6382	0.5495	0.8003	0.4098
**GRM4**	Q14833	PEC50	636	15.2887	0.6213	0.6098	0.7969	0.3928
**GRM5**	P41594	PIC50	1209	16.7918	0.7967	0.7932	0.893	0.5682

**GRM5**	P41594	PEC50	1450	15.0685	0.726	0.5388	0.8526	0.3761
**GRM5**	P41594	PKI	339	5.6975	0.7589	0.7653	0.873	0.5395
**GRM7**	Q14831	PEC50	99	0	0.559	0.7293	0.7641	0.5634
**GSK3A**	P49840	PIC50	338	19.8824	0.4509	0.6126	0.6789	0.3076
**GSK3B**	P49841	PIC50	1537	10.3026	0.6047	0.6726	0.7786	0.464
**GSK3B**	P49841	PKI	667	10.8836	0.5624	0.7729	0.7523	0.5232
**GYS1**	P13807	PIC50	232	19.3331	0.7539	0.3758	0.8712	0.2663
**GZMB**	P10144	PIC50	32	0	0.6515	0.7387	0.8215	0.4491
**HCRTR1**	O43613	PIC50	2152	13.4387	0.6564	0.6179	0.8118	0.4555
**HCRTR1**	O43613	PKI	988	20.1632	0.7615	0.6133	0.8737	0.4521
**HCRTR2**	O43614	PEC50	294	9.8001	0.7703	0.6878	0.8786	0.5267
**HCRTR2**	O43614	PKI	1115	10.8604	0.5838	0.7302	0.7657	0.554
**HCRTR2**	O43614	PIC50	2543	16.4901	0.6411	0.6124	0.8019	0.4388
**HDAC1**	Q13547	PIC50	2587	14.6547	0.6985	0.6834	0.8365	0.5027
**HDAC10**	Q969S8	PIC50	136	6.4761	0.504	0.8633	0.72	0.6563
**HDAC2**	Q92769	PIC50	902	13.3627	0.639	0.7487	0.8012	0.5427
**HDAC3**	O15379	PIC50	735	13.2434	0.7202	0.7297	0.8496	0.5329
**HDAC6**	Q9UBN7	PIC50	2342	11.4994	0.6736	0.5374	0.8223	0.3704
**HDAC8**	Q9BY41	PIC50	765	14.763	0.6841	0.5908	0.8281	0.4141
**HIF1A**	Q16665	PIC50	139	13.2379	0.5537	0.7073	0.7536	0.507
**HK2**	P52789	PIC50	77	19.25	0.4314	0.7381	0.6834	0.5943
**HMGCR**	P04035	PIC50	91	0	0.524	1.2246	0.7331	0.7368
**HPGD**	P15428	PIC50	180	10.0001	0.8361	0.3257	0.9153	0.1821
**HPGDS**	O60760	PIC50	733	9.245	0.7504	0.5071	0.8671	0.3692
**HPN**	P05981	PIC50	71	0	0.5228	0.6629	0.7535	0.495
**HRH1**	P35367	PKI	534	8.99	0.6839	0.8028	0.8283	0.5604
**HRH1**	P35367	PIC50	203	0	0.7222	0.6666	0.8532	0.4224
**HRH3**	Q9Y5N1	PIC50	440	8.8887	0.7602	0.5987	0.8729	0.4316
**HRH3**	Q9Y5N1	PKI	3060	12.4588	0.7544	0.6102	0.8696	0.4272
**HRH3**	Q9Y5N1	PEC50	248	20.667	0.7409	0.6722	0.8623	0.4389
**HRH4**	Q9H3N8	PKI	924	11.1592	0.519	0.7271	0.7226	0.5243
**HSD11B1**	P28845	PIC50	2278	13.0321	0.7676	0.7273	0.8772	0.5076
**HSD11B1**	P28845	PKI	655	9.161	0.7985	0.5872	0.895	0.3074

**HSD17B1**	P14061	PIC50	189	10.5	0.623	0.7234	0.8003	0.5456
**HSD17B2**	P37059	PIC50	186	10.3335	0.7247	0.5071	0.8555	0.3787
**HSD17B3**	P37058	PIC50	80	20	0.5393	0.8559	0.7546	0.6346
**HSP90AA1**	P07900	PIC50	570	7.1474	0.5968	0.6327	0.7747	0.4386
**HSP90AA1**	P07900	PKI	182	15.1667	0.6424	0.8114	0.8065	0.5773
**HSP90AB1**	P08238	PIC50	135	12.8573	0.5597	0.7546	0.7575	0.5267
**HTR1A**	P08908	PKI	2082	12.3928	0.656	0.7509	0.8105	0.5573
**HTR1A**	P08908	PIC50	521	8.0153	0.6216	0.7292	0.7916	0.534
**HTR1B**	P28222	PKI	321	3.3437	0.6166	0.7057	0.7874	0.5004
**HTR1D**	P28221	PKI	368	11.6824	0.7304	0.7042	0.8563	0.5144
**HTR2A**	P28223	PIC50	1036	9.4873	0.6292	0.8241	0.7967	0.5807
**HTR2A**	P28223	PKI	1778	15.5384	0.6482	0.6833	0.8065	0.4686
**HTR2B**	P41595	PIC50	163	20.3749	0.6575	0.7608	0.8151	0.5439
**HTR2C**	P28335	PEC50	983	17.0519	0.5184	0.8324	0.7213	0.596
**HTR2C**	P28335	PIC50	569	14.7793	0.6964	0.7149	0.8356	0.5195
**HTR2C**	P28335	PKI	1496	13.963	0.749	0.6706	0.866	0.4697
**HTR3A**	P46098	PIC50	127	7.0556	0.6821	0.9551	0.834	0.6769
**HTR3A**	P46098	PKI	130	6.1905	0.5279	1.059	0.7345	0.7498
**HTR4**	Q13639	PKI	534	5.394	0.7924	0.4872	0.8917	0.2554
**HTR4**	Q13639	PIC50	246	16.4002	0.588	0.5256	0.7711	0.366
**HTR4**	Q13639	PEC50	694	19.8285	0.8448	0.4526	0.9205	0.2534
**HTR5A**	P47898	PKI	564	9.1556	0.7207	0.4844	0.8503	0.3484
**HTR6**	P50406	PKI	2565	13.7523	0.7417	0.6238	0.8625	0.4361
**HTR6**	P50406	PIC50	610	11.4786	0.7336	0.6833	0.8575	0.4745
**HTR7**	P34969	PKI	989	12.1105	0.6178	0.7113	0.7874	0.5103
**HTRA1**	Q92743	PIC50	252	7.754	0.6619	0.4963	0.8178	0.3501
**ICAM1**	P05362	PIC50	86	10.7499	0.7181	0.7502	0.8565	0.567
**ICMT**	O60725	PIC50	102	10.1999	0.7617	0.7158	0.8811	0.5354
**IDE**	P14735	PIC50	100	0	0.4894	0.7423	0.72	0.5506
**IDH1**	O75874	PIC50	439	8.8687	0.6444	0.5259	0.8049	0.3838
**IDH2**	P48735	PIC50	64	0	0.7538	0.3852	0.8776	0.204
**IDO1**	P14902	PEC50	136	19.4284	0.6559	0.7655	0.8206	0.5703
**IDO1**	P14902	PIC50	2011	10.453	0.8238	0.6589	0.9081	0.4672

**IDO2**	Q6ZQW0	PIC50	77	0	0.5889	0.5235	0.7813	0.4008
**IGF1R**	P08069	PIC50	1697	15.8546	0.8671	0.6046	0.9314	0.41
**IKBKB**	O14920	PIC50	550	14.2857	0.8443	0.5678	0.9197	0.3809
**IL2**	P60568	PIC50	139	13.2379	0.8357	0.4788	0.9199	0.2712
**IMPDH2**	P12268	PIC50	193	4.8251	0.6781	0.5892	0.8279	0.4272
**IMPDH2**	P12268	PKI	123	0	0.8146	0.5575	0.9048	0.3832
**INSR**	P06213	PIC50	316	6.5835	0.794	0.6643	0.8927	0.4073
**IRAK4**	Q9NWZ3	PEC50	429	6.8095	0.5837	0.4295	0.7682	0.3334
**IRAK4**	Q9NWZ3	PKI	282	6.7142	0.6698	0.6711	0.8221	0.5101
**IRAK4**	Q9NWZ3	PIC50	1139	8.6881	0.6993	0.5398	0.8368	0.3481
**ITK**	Q08881	PIC50	382	15.0792	0.6891	0.5192	0.832	0.3265
**ITK**	Q08881	PKI	108	10.7997	0.6014	0.6956	0.7875	0.4924
**JAK1**	P23458	PEC50	97	9.6998	0.6351	0.3201	0.8029	0.1237
**JAK1**	P23458	PIC50	2127	11.6665	0.7085	0.6822	0.8422	0.4498
**JAK1**	P23458	PKI	590	9.8333	0.8023	0.4461	0.8964	0.3141
**JAK2**	O60674	PEC50	86	0	0.6683	0.2525	0.8314	0.1121
**JAK2**	O60674	PKI	744	8.0434	0.7652	0.609	0.8754	0.4267
**JAK2**	O60674	PIC50	3069	16.4418	0.6417	0.6666	0.8021	0.4595
**JAK3**	P52333	PEC50	48	0	0.5053	0.2046	0.7252	6.84E-02
**JAK3**	P52333	PIC50	1822	11.6792	0.7436	0.7019	0.8633	0.4749
**JAK3**	P52333	PKI	502	20.0803	0.876	0.6067	0.9365	0.3817
**KAT2B**	Q92831	PIC50	47	0	0.485	0.7001	0.755	0.539
**KAT6A**	Q92794	PIC50	344	17.3444	0.7675	0.5685	0.8775	0.4185
**KCNA3**	P22001	PIC50	240	0	0.5518	0.5121	0.7473	0.3568
**KCNH2**	Q12809	PIC50	2816	14.6224	0.5187	0.6636	0.722	0.4719
**KCNJ1**	P48048	PIC50	689	4.3425	0.5496	0.6416	0.7452	0.4358
**KCNK2**	O95069	PIC50	152	12.6666	0.5159	0.3926	0.7319	0.2721
**KCNK9**	Q9NPC2	PIC50	83	10.3752	0.4959	0.7369	0.7212	0.5674
**KCNMA1**	Q12791	PIC50	154	0	0.9252	0.6122	0.9632	0.3372
**KCNN4**	O15554	PIC50	61	0	0.5521	0.63	0.7658	0.4736
**KCNQ1**	P51787	PEC50	99	0	0.5778	0.5471	0.7753	0.4016
**KDM1A**	O60341	PKI	120	9.9998	0.5194	0.8504	0.732	0.6489

**KDM1A**	O60341	PIC50	618	13.2907	0.7636	0.5348	0.8747	0.3482
**KDM4A**	O75164	PIC50	66	0	0.5833	0.6237	0.7853	0.4316
**KDM4C**	Q9H3R0	PIC50	155	6.4583	0.6189	0.5834	0.7958	0.412
**KDM5A**	P29375	PIC50	92	0	0.6401	0.5497	0.808	0.3444
**KDM5B**	Q9UGL1	PIC50	167	0	0.6651	0.4863	0.822	0.3377
**KDR**	P35968	PIC50	5102	15.8887	0.7312	0.7308	0.8565	0.4893
**KDR**	P35968	PKI	414	14.7855	0.638	0.8769	0.8013	0.654
**KHK**	P50053	PIC50	132	0	0.5408	0.674	0.7456	0.5091
**KIF11**	P52732	PIC50	285	3.3928	0.715	0.7394	0.8481	0.5551
**KIT**	P10721	PIC50	1162	14.8084	0.7914	0.6085	0.8902	0.4168
**KLK1**	P06870	PIC50	314	13.0835	0.8662	0.5239	0.932	0.3842
**KLK7**	P49862	PIC50	113	9.4167	0.6431	0.7424	0.8123	0.5182
**KLKB1**	P03952	PIC50	1025	6.6993	0.7336	0.6095	0.8571	0.4198
**KLKB1**	P03952	PKI	515	11.8849	0.7444	0.7021	0.8646	0.5015
**KRAS**	P01116	PIC50	573	17.9626	0.8407	0.6941	0.9173	0.4686
**LCK**	P06239	PIC50	856	10.5389	0.7298	0.7079	0.8553	0.51
**LDHA**	P00338	PIC50	236	7.8668	0.729	0.5514	0.8598	0.3809
**LDHA**	P00338	PKI	100	20	0.7564	1.1471	0.8766	0.7784
**LGALS1**	P09382	PIC50	290	9.6665	0.7431	0.3962	0.8643	0.3055
**LGALS3**	P17931	PKI	84	20.9996	0.8676	0.6099	0.9353	0.4462
**LGALS3**	P17931	PIC50	522	12.0462	0.8167	0.4037	0.905	0.2981
**LIMK1**	P53667	PIC50	75	18.7498	0.4826	0.7575	0.7138	0.5387
**LIMK2**	P53671	PIC50	59	19.6668	0.5415	0.7817	0.7613	0.5991
**LIPC**	P11150	PIC50	136	12.9523	0.5333	0.6572	0.7401	0.5069
**LIPG**	Q9Y5X9	PIC50	305	13.5557	0.6514	0.5876	0.8107	0.4245
**LPAR1**	Q92633	PIC50	1189	17.6335	0.6499	0.5673	0.8068	0.3984
**LPAR2**	Q9HBW0	PIC50	68	22.6665	0.8368	0.5125	0.9184	0.3382
**LRRK2**	Q5S007	PKI	277	3.2976	0.6578	0.6659	0.815	0.4793
**LSS**	P48449	PIC50	337	19.8231	0.4687	0.5012	0.6903	0.2331
**LTA4H**	P09960	PIC50	724	14.3651	0.7347	0.7141	0.8586	0.51
**LTB4R**	Q15722	PIC50	210	14.3179	0.428	0.8802	0.6701	0.6577
**LTC4S**	Q16873	PIC50	83	20.7503	0.5051	0.6167	0.7301	0.4597
**MALT1**	Q9UDY8	PIC50	151	18.8746	0.5815	0.6878	0.7722	0.4556

**MAOA**	P21397	PIC50	973	9.4061	0.4714	0.8099	0.6901	0.584
**MAOA**	P21397	PKI	239	3.9832	0.5388	1.027	0.7405	0.7003
**MAOB**	P27338	PKI	236	15.7337	0.5573	0.9791	0.7524	0.7019
**MAOB**	P27338	PIC50	1680	14.1177	0.6659	0.8863	0.8172	0.6521
**MAP2K1**	Q02750	PIC50	536	14.438	0.6991	0.6656	0.8374	0.448
**MAP3K12**	Q12852	PIC50	201	20.1005	0.8688	0.2356	0.9351	0.1005
**MAP3K12**	Q12852	PKI	256	15.7541	0.5203	0.5718	0.7276	0.4274
**MAP3K5**	Q99683	PIC50	206	0	0.6412	0.6516	0.8109	0.4694
**MAP3K8**	P41279	PIC50	892	8.81	0.709	0.5528	0.843	0.4004
**MAP4K1**	Q92918	PIC50	789	15.173	0.9635	0.5715	0.9817	0.3491
**MAP4K1**	Q92918	PKI	101	10.1	0.5148	0.8343	0.7428	0.6049
**MAPK1**	P28482	PIC50	1277	17.651	0.7299	0.7071	0.8551	0.4946
**MAPK1**	P28482	PKI	93	18.6003	0.6254	0.9249	0.8014	0.6743
**MAPK10**	P53779	PIC50	424	17.6666	0.6811	0.7005	0.8271	0.518
**MAPK11**	Q15759	PIC50	106	0	0.6342	0.7134	0.8023	0.4636
**MAPK14**	Q16539	PIC50	2634	15.0604	0.7084	0.7341	0.8428	0.5358
**MAPK14**	Q16539	PKI	185	5.1388	0.5632	0.8936	0.7561	0.6839
**MAPK8**	P45983	PIC50	548	11.0707	0.587	0.6938	0.7682	0.4984
**MAPK9**	P45984	PIC50	340	11.4287	0.5843	0.6561	0.7692	0.4716
**MAPKAPK2**	P49137	PIC50	255	7.8461	0.5896	0.7602	0.7731	0.5869
**MAS1**	P04201	PIC50	63	0	0.8052	0.7032	0.9083	0.4196
**MBOAT4**	Q96T53	PIC50	151	12.5831	0.6334	0.6112	0.8128	0.454
**MC1R**	Q01726	PKI	303	16.8333	0.8045	0.723	0.8983	0.4943
**MC1R**	Q01726	PIC50	296	16.4447	0.7116	0.5297	0.8466	0.3522
**MC1R**	Q01726	PEC50	365	17.381	0.729	0.7854	0.8564	0.5931
**MC3R**	P41968	PKI	424	20.1904	0.8121	0.5895	0.9026	0.4239
**MC3R**	P41968	PEC50	231	15.4	0.6763	0.7049	0.8258	0.5166
**MC4R**	P32245	PKI	992	14.8802	0.7576	0.6635	0.8712	0.493
**MC4R**	P32245	PEC50	818	14.9633	0.783	0.7001	0.8854	0.4974
**MC4R**	P32245	PIC50	735	9.2703	0.8045	0.6546	0.8975	0.4352
**MC5R**	P33032	PKI	261	8.0308	0.6841	0.7307	0.8309	0.5241
**MC5R**	P33032	PEC50	123	10.2501	0.5092	0.9788	0.7357	0.7138
**MC5R**	P33032	PIC50	119	19.833	0.4809	0.6702	0.7016	0.4578

**MCHR1**	Q99705	PIC50	1894	5.2466	0.7301	0.8739	0.8549	0.5958
**MCHR1**	Q99705	PKI	746	12.0974	0.6608	0.6571	0.8139	0.4794
**MCL1**	Q07820	PIC50	936	15.7224	0.9306	0.5056	0.9648	0.3572
**MCL1**	Q07820	PKI	300	16.6667	0.8307	0.6019	0.9126	0.4392
**MDM2**	Q00987	PIC50	1084	11.8621	0.9025	0.6015	0.9503	0.414
**MDM2**	Q00987	PKI	154	6.4165	0.5849	0.9664	0.7733	0.7046
**MDM4**	O15151	PIC50	207	15.5247	0.7097	0.518	0.8477	0.3026
**MELK**	Q14680	PIC50	235	15.6664	0.6521	0.4945	0.8118	0.3237
**MEN1**	O00255	PIC50	71	17.7494	0.7217	0.7852	0.8568	0.5733
**MERTK**	Q12866	PIC50	156	6.4999	0.6599	0.3617	0.8134	0.2095
**MET**	P08581	PKI	260	4	0.5903	0.981	0.7732	0.7119
**MET**	P08581	PIC50	1629	7.3131	0.6983	0.5562	0.8375	0.3798
**METAP2**	P50579	PIC50	667	9.3288	0.8788	0.5269	0.9379	0.3291
**MGAT2**	Q10469	PIC50	441	13.3637	0.5208	0.6197	0.7245	0.4819
**MGLL**	Q99685	PIC50	946	14.8306	0.6962	0.5804	0.8351	0.3976
**MKNK1**	Q9BUB5	PIC50	878	16.6289	0.7659	0.515	0.876	0.3802
**MKNK2**	Q9HBH9	PIC50	508	6.096	0.7414	0.4837	0.8628	0.3273
**MLYCD**	O95822	PIC50	84	10.4998	0.6351	0.6811	0.808	0.5154
**MME**	P08473	PIC50	239	3.9832	0.7983	0.961	0.8952	0.6367
**MMP1**	P03956	PIC50	1055	15.1664	0.5724	0.8391	0.758	0.5788
**MMP1**	P03956	PKI	430	15.2022	0.6975	0.7152	0.8367	0.4594
**MMP12**	P39900	PIC50	493	17.748	0.8209	0.6332	0.9068	0.4115
**MMP13**	P45452	PIC50	2195	11.3376	0.7236	0.7709	0.8509	0.53
**MMP13**	P45452	PKI	598	14.9499	0.7858	0.6862	0.8873	0.4946
**MMP14**	P50281	PIC50	473	8.7595	0.6722	0.7804	0.8226	0.5541
**MMP2**	P08253	PIC50	2297	12.5923	0.7902	0.7961	0.8896	0.571
**MMP2**	P08253	PKI	707	18.7571	0.7172	0.7786	0.8479	0.5556
**MMP3**	P08254	PIC50	846	9.4788	0.61	0.8169	0.7815	0.537
**MMP3**	P08254	PKI	327	14.598	0.6891	0.8089	0.8349	0.5908
**MMP7**	P09237	PIC50	174	12.8892	0.6642	0.7546	0.8212	0.5696
**MMP8**	P22894	PIC50	443	4.4748	0.7346	0.7603	0.8585	0.5541
**MMP8**	P22894	PKI	231	11.55	0.7512	0.7286	0.869	0.4775
**MMP9**	P14780	PIC50	1728	8.6111	0.6929	0.8553	0.8332	0.5819

**MMP9**	P14780	PKI	759	5.3263	0.6979	0.8164	0.8364	0.598
**MPL**	P40238	PEC50	94	18.7996	0.5344	0.7195	0.747	0.5491
**MRGPRX2**	Q96LB1	PIC50	163	6.7916	0.4636	0.6093	0.6864	0.3979
**MST1R**	Q04912	PIC50	126	13.9997	0.6578	0.6327	0.8223	0.4456
**MTNR1A**	P48039	PKI	277	9.8927	0.5516	0.8848	0.7477	0.6673
**MTOR**	P42345	PKI	223	15.2047	0.8126	0.6778	0.9034	0.4938
**MTOR**	P42345	PIC50	2466	20.049	0.8366	0.8501	0.915	0.538
**MYC**	P01106	PIC50	289	10.3216	0.7872	0.4877	0.8894	0.3547
**NAAA**	Q02083	PIC50	103	10.3003	0.6499	0.7029	0.8132	0.5551
**NAMPT**	P43490	PEC50	130	12.3809	0.7645	0.47	0.8806	0.3453
**NAMPT**	P43490	PIC50	1665	18.8443	0.8375	0.5582	0.916	0.368
**NEK1**	Q96PY6	PIC50	64	21.3333	0.8113	0.4319	0.9111	0.3152
**NFKB1**	P19838	PIC50	135	19.2859	0.6793	0.6345	0.8303	0.4414
**NLRP3**	Q96P20	PEC50	390	19.4998	0.6555	0.4998	0.8129	0.3835
**NLRP3**	Q96P20	PIC50	84	10.4998	0.5878	0.646	0.7853	0.4655
**NMBR**	P28336	PKI	31	0	0.5409	0.9346	0.7976	0.7051
**NMT1**	P30419	PIC50	235	3.9166	0.5761	1.0579	0.7658	0.7715
**NNMT**	P40261	PIC50	36	17.9999	0.5475	0.8506	0.7891	0.6647
**NOS1**	P29475	PIC50	283	3.3691	0.5012	0.7942	0.7166	0.5602
**NOS2**	P35228	PIC50	333	16.7899	0.6463	0.8538	0.8074	0.6202
**NOTCH1**	P46531	PIC50	67	0	0.8724	0.5238	0.9378	0.3418
**NOTUM**	Q6P988	PIC50	87	10.8748	0.7728	0.5661	0.8834	0.3623
**NOX4**	Q9NPH5	PIC50	156	12.9998	0.758	0.3995	0.8747	0.287
**NPBWR1**	P48145	PIC50	117	19.5	0.4606	0.5311	0.6946	0.4052
**NPSR1**	Q6W5P4	PIC50	372	16.7821	0.6719	0.5064	0.8221	0.3815
**NPY1R**	P25929	PIC50	175	19.4446	0.8094	0.7028	0.902	0.4779
**NPY1R**	P25929	PKI	369	20.5002	0.7655	0.6219	0.8775	0.372
**NPY2R**	P49146	PIC50	225	12.2729	0.6438	0.5991	0.8082	0.4458
**NPY4R**	P50391	PEC50	166	20.7503	0.4011	0.6934	0.6424	0.5377
**NPY5R**	Q15761	PKI	613	16.4784	0.8403	0.592	0.9174	0.3579
**NPY5R**	Q15761	PIC50	930	7.2901	0.8138	0.7995	0.9025	0.5305
**NR1H2**	P55055	PIC50	630	16.6586	0.712	0.8217	0.8452	0.5728
**NR1H2**	P55055	PKI	176	4.8889	0.6635	0.5525	0.8233	0.4164

**NR1H2**	P55055	PEC50	761	17.3561	0.7745	0.6187	0.8807	0.4497
**NR1H3**	Q13133	PIC50	430	17.3739	0.7065	0.7768	0.842	0.5658
**NR1H3**	Q13133	PEC50	536	7.219	0.7791	0.6549	0.8836	0.4678
**NR1H4**	Q96RI1	PIC50	307	3.4111	0.7423	0.8642	0.864	0.5932
**NR1H4**	Q96RI1	PEC50	1323	10.7939	0.7532	0.5466	0.8684	0.3852
**NR1I2**	O75469	PEC50	163	6.7916	0.4401	0.6316	0.6718	0.4629
**NR3C1**	P04150	PKI	1035	9.478	0.5732	0.6824	0.7582	0.5101
**NR3C1**	P04150	PIC50	1540	6.4516	0.7349	0.7836	0.8579	0.5487
**NR3C1**	P04150	PEC50	449	13.6058	0.663	0.6919	0.8159	0.4953
**NR3C2**	P08235	PIC50	440	11.1109	0.5615	0.708	0.7549	0.5154
**NT5E**	P21589	PIC50	256	19.6926	0.826	0.4412	0.9095	0.2396
**NTRK1**	P04629	PIC50	1632	12.6649	0.7502	0.6256	0.8665	0.4149
**NTRK2**	Q16620	PIC50	365	14.4842	0.7416	0.8258	0.8632	0.5316
**NTRK3**	Q16288	PIC50	137	19.5713	0.8376	0.6448	0.9167	0.4137
**NTSR1**	P30989	PEC50	105	20.9996	0.8199	0.5676	0.9121	0.3977
**NTSR1**	P30989	PIC50	60	0	0.5424	0.819	0.7597	0.4815
**NTSR1**	P30989	PKI	88	11.0001	0.5807	0.9904	0.7724	0.744
**NUDT1**	P36639	PIC50	51	16.9999	0.6647	0.9323	0.8223	0.6698
**OPRD1**	P41143	PIC50	495	9.9	0.6369	0.8582	0.8035	0.63
**OPRD1**	P41143	PKI	1474	11.6768	0.8246	0.7359	0.9084	0.5144
**OPRD1**	P41143	PEC50	513	5.9193	0.6193	0.4945	0.7883	0.2702
**OPRK1**	P41145	PIC50	574	7.1974	0.7213	0.7062	0.8504	0.5035
**OPRK1**	P41145	PKI	1559	10.9605	0.6667	0.803	0.8173	0.6079
**OPRK1**	P41145	PEC50	754	18.5192	0.7745	0.5954	0.8806	0.3295
**OPRL1**	P41146	PKI	1038	10.4562	0.6044	0.6428	0.7793	0.4737
**OPRL1**	P41146	PIC50	363	14.4046	0.6297	0.7335	0.7964	0.5167
**OPRM1**	P35372	PIC50	549	9.2426	0.7051	0.7304	0.8408	0.5162
**OPRM1**	P35372	PKI	2371	10.598	0.7643	0.6976	0.8752	0.4812
**OPRM1**	P35372	PEC50	797	12.4533	0.7163	0.5994	0.8471	0.3628
**OXTR**	P30559	PEC50	121	10.0833	0.5104	1.0046	0.7352	0.7524
**OXTR**	P30559	PIC50	319	9.9686	0.7551	0.7739	0.8703	0.4069
**OXTR**	P30559	PKI	303	0	0.8091	0.5952	0.9008	0.3702
**P2RX2**	Q9UBL9	PIC50	83	0	0.6	0.4982	0.7914	0.4024

**P2RX3**	P56373	PIC50	2323	14.3664	0.6727	0.4782	0.8216	0.3401
**P2RX7**	Q99572	PIC50	1973	7.1541	0.6041	0.5596	0.778	0.3809
**P2RY1**	P47900	PKI	235	3.9166	0.5314	0.5677	0.7349	0.3918
**P2RY1**	P47900	PIC50	92	9.2001	0.6728	0.9238	0.8284	0.7234
**P2RY12**	Q9H244	PKI	323	6.7291	0.7079	0.545	0.8436	0.4089
**P2RY12**	Q9H244	PIC50	481	4.0083	0.5378	0.7459	0.7356	0.5433
**PADI4**	Q9UM07	PIC50	1402	9.2992	0.6589	0.4778	0.8126	0.3374
**PAK1**	Q13153	PKI	122	10.1669	0.561	0.622	0.7566	0.401
**PAK4**	O96013	PKI	125	6.9444	0.4821	0.7095	0.7077	0.54
**PAK4**	O96013	PIC50	90	9.0001	0.7152	0.5499	0.8554	0.3543
**PARG**	Q86W56	PIC50	214	14.5906	0.7936	0.3845	0.8937	0.2765
**PARP1**	P09874	PEC50	198	4.95	0.6537	0.6904	0.8138	0.5322
**PARP1**	P09874	PIC50	1565	11.0031	0.6702	0.5971	0.8196	0.4293
**PARP1**	P09874	PKI	429	9.0794	0.6547	0.5734	0.8111	0.3991
**PARP2**	Q9UGN5	PIC50	136	12.9523	0.7741	0.6623	0.8829	0.4694
**PAWR**	Q96IZ0	PIC50	111	9.25	0.6291	0.6352	0.8081	0.4681
**PCSK9**	Q8NBP7	PIC50	162	0	0.4786	0.9986	0.6994	0.7985
**PDE10A**	Q9Y233	PKI	399	9.9751	0.6931	1.0437	0.8357	0.7513
**PDE10A**	Q9Y233	PIC50	1680	12.9412	0.6667	0.6211	0.8173	0.4379
**PDE11A**	Q9HCR9	PIC50	80	20	0.5516	0.7013	0.7581	0.5178
**PDE1A**	P54750	PIC50	33	0	0.754	0.832	0.8813	0.5634
**PDE1B**	Q01064	PIC50	292	9.7333	0.6485	0.462	0.808	0.273
**PDE2A**	O00408	PIC50	556	16.2469	0.765	0.6936	0.8769	0.513
**PDE2A**	O00408	PKI	460	17.778	0.7371	0.5406	0.8601	0.3961
**PDE3A**	Q14432	PIC50	279	16.6074	0.7126	0.6435	0.8482	0.455
**PDE3B**	Q13370	PIC50	302	20.1329	0.7485	0.5041	0.8676	0.3522
**PDE4A**	P27815	PIC50	536	5.4142	0.631	0.6929	0.7965	0.4827
**PDE4B**	Q07343	PIC50	1581	19.3876	0.8827	0.6479	0.9397	0.4323
**PDE4C**	Q08493	PIC50	128	0	0.4149	0.7843	0.6751	0.5565
**PDE4D**	Q08499	PIC50	683	20.088	0.7895	0.5598	0.8898	0.3206
**PDE5A**	O76074	PIC50	1671	16.2757	0.8587	0.6851	0.9273	0.4762
**PDE7A**	Q13946	PIC50	307	0	0.6863	0.7032	0.831	0.4892
**PDE7B**	Q9NP56	PIC50	216	9.8179	0.4297	0.5207	0.6709	0.4032

**PDE8B**	O95263	PIC50	68	22.6665	0.5016	0.7558	0.7491	0.5899
**PDE9A**	O76083	PIC50	396	7.425	0.6055	0.5811	0.7821	0.4088
**PDGFRA**	P16234	PIC50	681	10.0147	0.717	0.7625	0.8478	0.4638
**PDGFRB**	P09619	PIC50	1146	14.8603	0.6228	0.8916	0.7902	0.6201
**PDK1**	Q15118	PIC50	425	13.4924	0.7134	0.5808	0.846	0.3852
**PDK2**	Q15119	PIC50	331	8.3446	0.8076	0.4412	0.9005	0.3088
**PDPK1**	O15530	PIC50	338	8.521	0.6396	0.8379	0.803	0.6207
**PFKFB3**	Q16875	PIC50	189	5.25	0.8878	0.4249	0.9431	0.2224
**PGR**	P06401	PIC50	1099	6.3579	0.8433	0.727	0.9188	0.517
**PGR**	P06401	PKI	387	15.2762	0.7526	0.6214	0.8692	0.4669
**PGR**	P06401	PEC50	420	2.5	0.841	0.9306	0.9176	0.5812
**PHGDH**	O43175	PIC50	110	9.1666	0.6082	0.7902	0.7872	0.6004
**PI4KB**	Q9UBF8	PIC50	112	0	0.7569	0.6145	0.8743	0.4109
**PIK3C3**	Q8NEB9	PIC50	173	0	0.7244	0.4855	0.8553	0.3093
**PIK3CA**	P42336	PKI	629	14.047	0.8517	0.6379	0.9234	0.4772
**PIK3CA**	P42336	PIC50	2661	12.0802	0.7271	0.6366	0.854	0.4376
**PIK3CB**	P42338	PKI	205	15.3752	0.5184	0.7341	0.7273	0.5557
**PIK3CB**	P42338	PIC50	1160	12.1737	0.7741	0.6207	0.8808	0.4361
**PIK3CD**	O00329	PKI	273	3.9	0.5875	0.7226	0.7707	0.5285
**PIK3CD**	O00329	PIC50	1907	11.6214	0.759	0.6149	0.8717	0.4276
**PIK3CG**	P48736	PIC50	1455	13.0588	0.7541	0.5534	0.8691	0.3695
**PIK3CG**	P48736	PKI	193	19.3004	0.7823	0.6699	0.8876	0.489
**PIKFYVE**	Q9Y2I7	PIC50	117	9.75	0.5312	0.6332	0.7354	0.4853
**PIM1**	P11309	PKI	563	10.9676	0.8493	0.7725	0.9221	0.5633
**PIM1**	P11309	PIC50	1338	12.574	0.7252	0.6867	0.8522	0.4807
**PIM2**	Q9P1W9	PKI	166	0	0.5566	0.7882	0.7549	0.6018
**PIM2**	Q9P1W9	PIC50	857	16.4128	0.6582	0.6815	0.813	0.4949
**PIM3**	Q86V86	PIC50	395	14.8126	0.5575	0.6387	0.7492	0.4352
**PIM3**	Q86V86	PKI	175	14.5834	0.772	0.9291	0.8824	0.6873
**PIN1**	Q13526	PKI	52	17.3331	0.5415	0.9167	0.7546	0.6834
**PKN2**	Q16513	PIC50	78	0	0.6537	0.4159	0.8147	0.1884
**PLA2G1B**	P04054	PIC50	140	13.3331	0.6926	0.6689	0.8422	0.4498
**PLA2G2A**	P14555	PIC50	142	20.2855	0.6498	0.7723	0.8145	0.5783

**PLA2G2D**	Q9UNK4	PIC50	122	20.3339	0.5845	0.8153	0.7746	0.5891
**PLA2G7**	Q13093	PIC50	250	15.3846	0.839	0.5976	0.9173	0.4291
**PLAT**	P00750	PIC50	240	4.0001	0.8119	0.5721	0.9047	0.3681
**PLAU**	P00749	PIC50	131	12.4761	0.5029	0.8805	0.7256	0.6519
**PLAU**	P00749	PKI	322	13.4163	0.6485	0.7221	0.8082	0.511
**PLD1**	Q13393	PIC50	70	17.4997	0.6855	0.6784	0.8383	0.5365
**PLG**	P00747	PIC50	225	12.2729	0.7737	0.7162	0.8824	0.5063
**PLG**	P00747	PKI	252	15.5079	0.7398	0.8106	0.8642	0.5834
**PLK1**	P53350	PIC50	420	12.4998	0.7263	0.621	0.8533	0.3893
**PLK4**	O00444	PIC50	194	14.5497	0.7027	0.3913	0.8408	0.2095
**PNP**	P00491	PKI	99	19.8	0.5472	1.343	0.7542	0.9972
**PPARA**	Q07869	PIC50	683	15.7835	0.7291	0.4885	0.8548	0.2761
**PPARA**	Q07869	PEC50	1388	15.8053	0.6423	0.7995	0.8026	0.5592
**PPARD**	Q03181	PIC50	515	11.8849	0.5782	0.6452	0.7631	0.3931
**PPARD**	Q03181	PKI	58	19.3338	0.4984	0.5325	0.7199	0.2762
**PPARD**	Q03181	PEC50	961	11.5911	0.7228	0.7559	0.8511	0.5045
**PPARG**	P37231	PIC50	765	9.3947	0.7027	0.6193	0.8396	0.4235
**PPARG**	P37231	PEC50	1746	13.1881	0.632	0.7521	0.7965	0.5348
**PPIA**	P62937	PKI	107	0	0.8603	0.642	0.9319	0.4453
**PPIA**	P62937	PIC50	69	22.9996	0.6817	0.9105	0.8396	0.6743
**PRCP**	P42785	PIC50	128	14.2219	0.5516	0.8986	0.763	0.6896
**PREP**	P48147	PIC50	397	9.9251	0.7943	0.7696	0.8923	0.5527
**PRKAA1**	Q13131	PEC50	92	18.4001	0.5428	0.4868	0.7547	0.3689
**PRKAA2**	P54646	PEC50	187	10.3891	0.6171	0.6765	0.7936	0.5128
**PRKCA**	P17252	PIC50	312	16.2498	0.7288	0.7514	0.8561	0.5384
**PRKCB**	P05771	PIC50	152	12.6666	0.572	0.7912	0.7646	0.5645
**PRKCD**	Q05655	PIC50	144	6.8573	0.4415	0.7595	0.6771	0.5497
**PRKCQ**	Q04759	PIC50	607	8.4307	0.6312	0.6025	0.7964	0.4339
**PRKCQ**	Q04759	PKI	74	18.4993	0.4697	0.9823	0.702	0.7442
**PRKCZ**	Q05513	PIC50	68	0	0.5315	0.8232	0.7513	0.639
**PRKDC**	P78527	PIC50	436	8.8082	0.6873	0.7432	0.8306	0.5394
**PRMT1**	Q99873	PIC50	345	5.7983	0.6676	0.6311	0.8261	0.4455
**PRMT3**	O60678	PIC50	31	0	0.4234	0.7057	0.7007	0.5628

**PRMT5**	O14744	PIC50	148	0	0.5249	0.7057	0.7341	0.5167
**PRMT6**	Q96LA8	PIC50	161	6.7082	0.5378	0.5034	0.7395	0.3251
**PRMT8**	Q9NR22	PIC50	128	0	0.527	0.5021	0.7365	0.3353
**PRSS1**	P07477	PIC50	221	20.0904	0.7436	0.7848	0.8647	0.5692
**PRSS1**	P07477	PKI	579	13.3106	0.6945	0.8389	0.8345	0.5672
**PSMB2**	P49721	PIC50	72	0	0.5832	0.7392	0.7795	0.5769
**PSMB5**	P28074	PIC50	477	13.9127	0.6183	0.7497	0.7886	0.5214
**PSMB8**	P28062	PIC50	294	9.8001	0.7311	0.5826	0.8567	0.3708
**PSMB9**	P28065	PIC50	86	21.4998	0.5675	0.6289	0.7645	0.456
**PTGDR**	Q13258	PKI	110	0	0.6099	0.8455	0.7918	0.6371
**PTGDR2**	Q9Y5Y4	PIC50	1367	6.7011	0.6276	0.7245	0.7928	0.5186
**PTGDR2**	Q9Y5Y4	PKI	721	7.153	0.6927	0.6003	0.8332	0.4247
**PTGDS**	P41222	PIC50	125	6.9444	0.6511	0.5665	0.816	0.4303
**PTGER1**	P34995	PKI	111	0	0.7063	0.8999	0.8472	0.6413
**PTGER1**	P34995	PIC50	453	6.5651	0.757	0.5519	0.8712	0.3378
**PTGER2**	P43116	PKI	241	0	0.6813	0.7068	0.8286	0.5041
**PTGER2**	P43116	PIC50	928	14.8651	0.6924	0.475	0.8335	0.3524
**PTGER2**	P43116	PEC50	194	14.5497	0.5696	0.8519	0.7623	0.6281
**PTGER3**	P43115	PKI	154	12.833	0.7355	0.7797	0.8613	0.5723
**PTGER3**	P43115	PIC50	540	7.2729	0.5215	0.6995	0.724	0.4345
**PTGER4**	P35408	PKI	245	8.1667	0.6557	0.7134	0.8119	0.5131
**PTGER4**	P35408	PIC50	889	10.1025	0.6458	0.4908	0.8046	0.3596
**PTGER4**	P35408	PEC50	214	9.7271	0.6612	0.9739	0.8175	0.6719
**PTGES**	O14684	PIC50	1147	1.7498	0.7622	0.5564	0.8735	0.4065
**PTGIR**	P43119	PKI	78	9.75	0.4292	1.0073	0.693	0.7016
**PTGIR**	P43119	PEC50	75	0	0.6719	0.8135	0.8302	0.5139
**PTGS2**	P35354	PIC50	1677	11.7439	0.5418	0.8663	0.7369	0.6355
**PTH1R**	Q03431	PKI	126	0	0.7444	0.5205	0.8684	0.3896
**PTK2**	Q05397	PIC50	1191	16.5418	0.923	0.4577	0.9608	0.2916
**PTK2**	Q05397	PKI	94	18.7996	0.6122	0.9007	0.8007	0.7005
**PTK2B**	Q14289	PIC50	72	17.9999	0.7924	0.526	0.8945	0.3861
**PTPN1**	P18031	PIC50	1542	17.4418	0.8341	0.6243	0.9135	0.4134
**PTPN1**	P18031	PKI	262	16.1231	0.661	0.757	0.8171	0.5545

**PTPN11**	Q06124	PIC50	420	4.9999	0.844	0.5343	0.9195	0.3712
**PTPN2**	P17706	PIC50	250	19.2308	0.7161	0.4974	0.8494	0.335
**PTPRB**	P23467	PIC50	75	0	0.7604	1.0818	0.8789	0.8055
**PYGL**	P06737	PIC50	194	9.6998	0.6208	0.7968	0.7917	0.5487
**QPCT**	Q16769	PKI	133	19.0003	0.7458	0.6548	0.8717	0.4422
**QPCT**	Q16769	PIC50	467	13.5362	0.8408	0.5814	0.9175	0.3551
**RAF1**	P04049	PIC50	581	18.365	0.8449	0.5489	0.9198	0.3541
**RARG**	P13631	PIC50	40	0	0.4463	0.6426	0.6962	0.3422
**REN**	P00797	PIC50	3279	13.0264	0.7213	0.9515	0.8499	0.6289
**RET**	P07949	PIC50	1380	17.857	0.8431	0.5403	0.9185	0.3444
**RIPK1**	Q13546	PIC50	866	5.9235	0.7974	0.8331	0.8935	0.5509
**RIPK1**	Q13546	PKI	317	6.6043	0.5598	0.5971	0.7518	0.446
**RIPK2**	O43353	PIC50	148	0	0.6339	0.7655	0.8012	0.5073
**ROCK1**	Q13464	PIC50	522	4.0154	0.6983	0.785	0.8373	0.5408
**ROCK2**	O75116	PIC50	762	10.6948	0.7566	0.7994	0.8706	0.575
**RORC**	P51449	PIC50	3362	13.4406	0.6588	0.6091	0.8133	0.4339
**RORC**	P51449	PKI	594	18.15	0.6885	0.7149	0.8317	0.5263
**RORC**	P51449	PEC50	467	4.5121	0.421	0.6193	0.6543	0.4582
**ROS1**	P08922	PIC50	70	0	0.5658	0.8425	0.7691	0.5892
**RPS6KA3**	P51812	PIC50	160	20	0.6479	0.7634	0.8086	0.5397
**RPS6KB1**	P23443	PIC50	427	11.2965	0.5804	0.757	0.7717	0.5548
**RXRA**	P19793	PIC50	58	0	0.7944	0.538	0.899	0.3986
**RXRA**	P19793	PKI	81	20.2503	0.5735	0.8117	0.7743	0.6291
**RXRA**	P19793	PEC50	126	20.9996	0.5122	0.9146	0.7363	0.6646
**RXRB**	P28702	PEC50	58	0	0.4684	0.8015	0.7271	0.599
**S1PR1**	P21453	PEC50	1540	12.258	0.63	0.8415	0.7943	0.5843
**S1PR1**	P21453	PIC50	365	5.7937	0.6828	0.7066	0.828	0.4859
**S1PR2**	O95136	PIC50	110	9.1666	0.555	0.6881	0.7596	0.5287
**S1PR3**	Q99500	PIC50	187	5.1945	0.5575	0.741	0.7532	0.5441
**S1PR4**	O95977	PEC50	103	20.6005	0.5545	0.7775	0.7528	0.5502
**S1PR4**	O95977	PIC50	91	9.1	0.5045	0.5751	0.7262	0.4487
**S1PR5**	Q9H228	PIC50	118	0	0.8508	0.5674	0.9262	0.3651
**S1PR5**	Q9H228	PEC50	273	11.7	0.7489	0.6044	0.8666	0.4253

**SCD**	O00767	PIC50	528	11.0771	0.6503	0.8762	0.8115	0.6641
**SCN10A**	Q9Y5Y9	PIC50	984	10.0409	0.6902	0.6504	0.8316	0.4716
**SCN2A**	Q99250	PIC50	328	8.7859	0.6765	0.8547	0.8253	0.6192
**SCN3A**	Q9NY46	PIC50	166	13.8336	0.7598	0.5428	0.8746	0.3354
**SCN4A**	P35499	PIC50	250	7.6923	0.6923	0.9242	0.8363	0.6839
**SCN5A**	Q14524	PIC50	798	8.7282	0.7454	0.6534	0.8649	0.4516
**SCN8A**	Q9UQD0	PIC50	234	15.5997	0.4937	0.7721	0.7103	0.5732
**SCN9A**	Q15858	PIC50	3894	11.5206	0.7115	0.5927	0.8439	0.4341
**SELE**	P16581	PIC50	87	10.8748	0.8037	0.834	0.9031	0.6343
**SELP**	P16109	PIC50	91	0	0.5046	0.8954	0.7284	0.6612
**SERPINE1**	P05121	PIC50	214	0	0.568	0.6987	0.7649	0.4962
**SGK1**	O00141	PIC50	253	11.6768	0.634	0.7821	0.7999	0.565
**SIGMAR1**	Q99720	PKI	547	9.2086	0.585	0.6521	0.7666	0.4603
**SIGMAR1**	Q99720	PIC50	223	10.1365	0.5445	0.666	0.7435	0.4925
**SIRT2**	Q8IXJ6	PIC50	385	20.2634	0.5945	0.6431	0.7733	0.4717
**SLC10A2**	Q12908	PIC50	217	14.7956	0.6618	0.6622	0.8175	0.4947
**SLC18A2**	Q05940	PKI	68	0	0.8906	0.4039	0.9463	0.2124
**SLC1A2**	P43004	PIC50	52	0	0.7002	0.7686	0.8521	0.5746
**SLC1A3**	P43003	PIC50	57	18.9999	0.472	0.9222	0.7116	0.7366
**SLC27A4**	Q6P1M0	PIC50	62	0	0.7119	0.3497	0.8827	0.1548
**SLC29A2**	Q14542	PIC50	129	7.1666	0.7919	0.7608	0.8931	0.5287
**SLC2A1**	P11166	PIC50	224	20.3638	0.6374	0.599	0.8038	0.422
**SLC34A1**	Q06495	PIC50	185	5.1388	0.6074	0.6542	0.785	0.5168
**SLC34A2**	O95436	PIC50	201	10.0503	0.8034	0.3632	0.8996	0.2522
**SLC40A1**	Q9NP59	PEC50	234	7.7998	0.6376	0.5826	0.808	0.4354
**SLC5A1**	P13866	PIC50	603	15.0754	0.7225	0.591	0.8514	0.4269
**SLC5A2**	P31639	PIC50	734	9.2576	0.6331	0.6402	0.7971	0.462
**SLC5A2**	P31639	PEC50	51	0	0.6529	0.5931	0.818	0.4239
**SLC5A5**	Q92911	PIC50	68	22.6665	0.501	0.4995	0.7473	0.3612
**SLC6A2**	P23975	PEC50	79	0	0.5764	0.82	0.7853	0.5817
**SLC6A2**	P23975	PKI	1063	14.3264	0.6684	0.6231	0.818	0.4055
**SLC6A2**	P23975	PIC50	804	13.8191	0.5732	0.7049	0.7581	0.502
**SLC6A3**	Q01959	PIC50	600	5	0.479	0.7861	0.6985	0.5802

**SLC6A3**	Q01959	PKI	752	10.5543	0.65	0.6226	0.8074	0.4336
**SLC6A4**	P31645	PKI	1497	13.3067	0.7141	0.6914	0.8456	0.4584
**SLC6A4**	P31645	PIC50	1542	11.6279	0.6169	0.5956	0.7863	0.4162
**SLC6A5**	Q9Y345	PIC50	188	10.4443	0.7629	0.6369	0.8827	0.3977
**SLC6A9**	P48067	PIC50	776	16.1663	0.6241	0.5899	0.7916	0.377
**SLC8A1**	P32418	PIC50	231	15.4	0.4349	0.4253	0.669	0.3079
**SLC9A1**	P19634	PIC50	131	18.7141	0.6116	0.7018	0.7923	0.4991
**SLC9A3**	P48764	PIC50	105	10.4998	0.4684	0.6067	0.7004	0.4151
**SMARCA4**	P51532	PIC50	120	9.9998	0.5797	0.6313	0.7716	0.4937
**SMO**	Q99835	PKI	114	9.4999	0.7246	0.3986	0.8862	0.1117
**SMYD3**	Q9H7B4	PIC50	415	9.881	0.8551	0.5512	0.9258	0.4229
**SOAT1**	P35610	PIC50	131	18.7141	0.6875	0.5977	0.835	0.4348
**SOAT2**	O75908	PIC50	84	20.9996	0.7621	0.7138	0.8813	0.5402
**SOS1**	Q07889	PKI	110	18.3332	0.8506	0.6423	0.9263	0.4496
**SOS1**	Q07889	PIC50	72	17.9999	0.7483	0.4723	0.8727	0.3262
**SPHK1**	Q9NYA1	PIC50	60	0	0.4889	0.9096	0.7306	0.6856
**SPR**	P35270	PIC50	455	6.5943	0.5363	0.7842	0.7363	0.5195
**SRC**	P12931	PIC50	2091	10.9055	0.7316	0.7483	0.8564	0.5236
**SRD5A1**	P18405	PKI	123	20.5002	0.6464	0.7017	0.8105	0.5179
**SRD5A2**	P31213	PIC50	113	9.4167	0.7351	0.8228	0.8598	0.4883
**SRD5A2**	P31213	PKI	75	0	0.5489	0.6906	0.7552	0.4862
**SSTR1**	P30872	PIC50	136	6.4761	0.6149	0.4812	0.7939	0.2435
**SSTR1**	P30872	PKI	155	19.3748	0.752	0.4365	0.8681	0.2296
**SSTR2**	P30874	PEC50	79	9.8752	0.6909	0.4758	0.8337	0.2861
**SSTR2**	P30874	PIC50	268	8.246	0.6643	0.5895	0.8172	0.2919
**SSTR2**	P30874	PKI	205	10.2501	0.802	0.5978	0.8985	0.3219
**SSTR3**	P32745	PKI	259	11.9539	0.6363	0.6772	0.8074	0.4677
**SSTR3**	P32745	PIC50	198	14.85	0.7101	0.449	0.8453	0.2552
**SSTR4**	P31391	PEC50	175	14.5834	0.5661	0.5994	0.7574	0.4257
**SSTR4**	P31391	PIC50	152	0	0.7048	0.4583	0.8419	0.2053
**SSTR4**	P31391	PKI	183	5.0833	0.7094	0.4993	0.845	0.2836
**SSTR5**	P35346	PKI	807	2.5218	0.764	1.0513	0.8746	0.6156
**SSTR5**	P35346	PIC50	344	5.7815	0.8043	0.7217	0.8979	0.5196

**ST14**	Q9Y5Y6	PKI	183	20.3333	0.7364	0.6839	0.8616	0.4111
**STAT3**	P40763	PIC50	236	11.8003	0.6153	0.5936	0.7894	0.4368
**STS**	P08842	PIC50	274	11.743	0.656	0.928	0.814	0.6484
**SYK**	P43405	PIC50	2246	12.9232	0.8154	0.5292	0.9033	0.3363
**TACR1**	P25103	PIC50	1317	11.5121	0.7715	0.6808	0.8787	0.4484
**TACR1**	P25103	PKI	1169	12.2683	0.7914	0.6887	0.8901	0.433
**TACR2**	P21452	PIC50	519	11.9768	0.7511	0.6527	0.8676	0.4006
**TACR2**	P21452	PKI	652	10.6385	0.8143	0.7252	0.9034	0.4894
**TACR3**	P29371	PEC50	37	0	0.6292	0.9748	0.8302	0.7464
**TACR3**	P29371	PIC50	444	15.6972	0.5388	0.7177	0.7386	0.4738
**TACR3**	P29371	PKI	813	9.9147	0.6231	0.5164	0.7896	0.3457
**TBK1**	Q9UHD2	PIC50	402	10.0503	0.6166	0.4946	0.7871	0.3359
**TBXA2R**	P21731	PKI	240	8.0001	0.5307	0.7831	0.7369	0.546
**TDO2**	P48775	PIC50	382	7.5396	0.9356	0.5448	0.9675	0.338
**TDP1**	Q9NUW8	PIC50	90	18.0002	0.4623	0.7521	0.7021	0.5319
**TDP2**	O95551	PIC50	99	19.8	0.5364	0.5678	0.7513	0.414
**TEK**	Q02763	PIC50	754	18.5192	0.7726	0.6591	0.8796	0.4239
**TGFBR1**	P36897	PIC50	448	13.576	0.758	0.5776	0.8715	0.3599
**TGM2**	P21980	PIC50	217	4.9319	0.4564	0.6331	0.6813	0.4569
**THRA**	P10827	PIC50	123	20.5002	0.6556	0.7373	0.8164	0.5585
**THRA**	P10827	PKI	96	0	0.5689	0.8388	0.7743	0.4553
**THRB**	P10828	PIC50	227	4.1274	0.6583	0.8862	0.8231	0.6391
**THRB**	P10828	PKI	103	10.3003	0.4427	0.8031	0.6927	0.4504
**TLR7**	Q9NYK1	PEC50	914	13.2461	0.8144	0.6065	0.9033	0.4497
**TLR7**	Q9NYK1	PIC50	2712	12.9245	0.7972	0.4942	0.8931	0.3602
**TLR8**	Q9NR97	PIC50	2277	13.4605	0.732	0.5253	0.8563	0.3778
**TLR8**	Q9NR97	PEC50	416	12.3808	0.8522	0.5425	0.9239	0.3999
**TLR9**	Q9NR96	PIC50	2488	13.2429	0.7537	0.4485	0.8684	0.3287
**TMPRSS15**	P98073	PKI	94	9.3998	0.6213	0.4	0.8058	0.2595
**TNF**	P01375	PIC50	985	17.0864	0.8701	0.7052	0.9336	0.4635
**TNF**	P01375	PKI	100	10	0.8144	0.6359	0.9114	0.4096
**TNK2**	Q07912	PIC50	148	0	0.4779	0.6275	0.7147	0.496
**TNK2**	Q07912	PKI	69	0	0.5251	0.867	0.7488	0.6731

**TNKS**	O95271	PIC50	264	20.3081	0.4478	0.7608	0.6769	0.5631
**TNKS2**	Q9H2K2	PIC50	268	8.246	0.5507	0.6877	0.7473	0.52
**TOP1**	P11387	PIC50	132	6.2858	0.5906	0.8127	0.7749	0.5702
**TPH1**	P17752	PIC50	467	9.0241	0.6136	0.4249	0.7855	0.2912
**TRIM24**	O15164	PIC50	111	9.25	0.8178	0.7345	0.9124	0.5336
**TRPA1**	O75762	PEC50	63	21	0.4977	1.0424	0.7354	0.8043
**TRPA1**	O75762	PIC50	1154	12.9758	0.6467	0.5615	0.8075	0.4196
**TRPM8**	Q7Z2W7	PIC50	619	9.9839	0.447	0.6883	0.671	0.4644
**TRPV1**	Q8NER1	PIC50	1731	14.78	0.5508	0.6899	0.7445	0.4983
**TRPV1**	Q8NER1	PKI	710	11.27	0.6021	0.6354	0.7775	0.444
**TRPV3**	Q8NET8	PIC50	306	16.9999	0.6784	0.4714	0.8276	0.3474
**TRPV4**	Q9HBA0	PIC50	652	16.7177	0.7504	0.6997	0.8675	0.4737
**TTK**	P33981	PIC50	945	13.7569	0.8421	0.4853	0.918	0.326
**TYK2**	P29597	PEC50	208	15.5998	0.5594	0.4204	0.753	0.2761
**TYK2**	P29597	PIC50	920	12.2224	0.6619	0.728	0.8147	0.4777
**TYK2**	P29597	PKI	498	7.9681	0.8786	0.4938	0.9376	0.3221
**TYMP**	P19971	PIC50	84	10.4998	0.507	0.647	0.7325	0.4411
**TYMS**	P04818	PKI	156	19.4998	0.7036	0.8517	0.8435	0.6064
**TYRO3**	Q06418	PIC50	136	6.4761	0.5366	0.4932	0.7366	0.3818
**USP7**	Q93009	PIC50	85	21.2504	0.7681	0.643	0.8848	0.4818
**UTS2R**	Q9UKP6	PIC50	429	15.8889	0.5495	0.5573	0.7556	0.2784
**UTS2R**	Q9UKP6	PKI	602	20.0662	0.7812	0.5451	0.8856	0.3398
**UTS2R**	Q9UKP6	PEC50	102	20.3998	0.7259	0.6291	0.8618	0.4622
**VCAM1**	P19320	PIC50	61	0	0.6626	0.8141	0.8281	0.6219
**VDR**	P11473	PIC50	91	9.1	0.843	0.603	0.9241	0.4368
**VNN1**	O95497	PIC50	143	13.6191	0.6513	0.558	0.8115	0.3574
**WDR5**	P61964	PKI	78	9.75	0.8047	0.8153	0.9038	0.6023
**WNT3**	P56703	PIC50	57	0	0.5423	0.7579	0.7619	0.5851
**XDH**	P47989	PIC50	120	0	0.7424	0.771	0.8659	0.5266
**XIAP**	P98170	PIC50	438	13.2727	0.7084	0.5935	0.8438	0.4194
**XIAP**	P98170	PKI	112	0	0.7297	0.7597	0.8592	0.559
**YES1**	P07947	PIC50	338	14.2017	0.607	0.5546	0.7888	0.3569

Table S3. Performance on the 18 targets selected for prospective validation.

Gene Name	Algorithm	Target Property	Uniprot ID	AD	N_All	N_In	r	RMSE	MAE
**ADORA2A**	GB	pKI	P29274	All	167	53	0.60	1.28	1.05
**ADORA2A**	GB	pKI	P29274	In	167	53	0.57	1.49	1.28
**CCR6**	GB	pIC50	P51684	All	488	94	0.28	0.57	0.45
**CCR6**	GB	pIC50	P51684	In	488	94	0.33	0.44	0.37
**CD38**	GB	pIC50	P28907	All	381	68	0.53	0.91	0.74
**CD38**	GB	pIC50	P28907	In	381	68	0.65	0.94	0.75
**CGAS**	GB	pIC50	Q8N884	All	665	60	0.40	0.99	0.85
**CGAS**	GB	pIC50	Q8N884	In	665	60	0.91	0.50	0.37
**GABRA5**	GB	pKI	P31644	All	679	94	0.42	0.69	0.55
**GABRA5**	GB	pKI	P31644	In	679	94	0.71	0.51	0.40
**GLP1R**	GB	pEC50	P43220	All	702	437	0.67	0.90	0.65
**GLP1R**	GB	pEC50	P43220	In	702	437	0.75	0.76	0.51
**GPBAR1**	GB	pEC50	Q8TDU6	All	386	71	0.05	0.90	0.73
**GPBAR1**	GB	pEC50	Q8TDU6	In	386	71	0.35	0.81	0.67
**HDAC6**	GB	pIC50	Q9UBN7	All	414	132	0.11	2.02	1.51
**HDAC6**	GB	pIC50	Q9UBN7	In	414	132	-0.04	1.60	1.02
**HPGDS**	GB	pIC50	O60760	All	531	111	0.56	0.76	0.57
**HPGDS**	GB	pIC50	O60760	In	531	111	0.74	0.38	0.27
**IDO1**	GB	pIC50	P14902	All	124	60	0.72	1.40	0.95
**IDO1**	GB	pIC50	P14902	In	124	60	0.73	1.07	0.66
**KLKB1**	GB	pIC50	P03952	All	746	141	0.40	0.95	0.76
**KLKB1**	GB	pIC50	P03952	In	746	141	0.70	0.84	0.65
**KRAS**	GB	pIC50	P01116	All	1077	107	0.60	0.97	0.81
**KRAS**	GB	pIC50	P01116	In	1077	107	0.70	0.96	0.76
**LTA4H**	GB	pIC50	P09960	All	82	57	0.61	0.48	0.34
**LTA4H**	GB	pIC50	P09960	In	82	57	0.72	0.42	0.31
**PPARA**	GB	pEC50	Q07869	All	84	50	0.20	1.36	1.02
**PPARA**	GB	pEC50	Q07869	In	84	50	0.17	1.35	1.03
**TLR7**	GB	pIC50	Q9NYK1	All	332	78	0.90	0.53	0.40
**TLR7**	GB	pIC50	Q9NYK1	In	332	78	0.94	0.47	0.33
**TLR8**	GB	pIC50	Q9NR97	All	275	66	0.84	0.83	0.65
**TLR8**	GB	pIC50	Q9NR97	In	275	66	0.89	0.77	0.61
**TLR9**	GB	pIC50	Q9NR96	All	260	92	0.84	0.42	0.34
**TLR9**	GB	pIC50	Q9NR96	In	260	92	0.82	0.43	0.33
**TRPV3**	GB	pIC50	Q8NET8	All	109	70	0.65	0.38	0.31
**TRPV3**	GB	pIC50	Q8NET8	In	109	70	0.69	0.37	0.29

Table S4. Summary of structural overlap analysis between test and training set compounds across selected targets for case study #1. For each target, the table reports the maximum pairwise Tanimoto similarity (mean, standard deviation, minimum, maximum, and percentiles) between test compounds and their closest analogues in the training set, along with the ratio of shared scaffolds using wireframe and Bemis–Murcko definitions. These metrics quantify the extent of chemical similarity and scaffold redundancy.

Gene Name	Maximum pairwise Tanimoto similarity against the training set						Ratio of shared scaffolds	
	**Mean**	**StdDev**	**Min**	**Max**	**Percentile25**	**Percentile90**	**Wireframe**	**Bemis-Murcko**
ADORA2A	0.62	0.12	0.32	0.86	0.52	0.76	0.33	0.07
CCR6	0.53	0.15	0.26	0.86	0.41	0.77	0.44	0.04
CD38	0.52	0.17	0.16	1.00	0.38	0.76	0.24	0.11
CGAS	0.44	0.15	0.19	0.91	0.34	0.65	0.17	0.09
GABRA5	0.54	0.13	0.26	0.89	0.47	0.73	0.26	0.10
GLP1R	0.76	0.11	0.43	0.93	0.69	0.89	0.30	0.14
GPBAR1	0.56	0.10	0.37	0.89	0.50	0.67	0.21	0.03
HDAC6	0.63	0.12	0.37	0.89	0.53	0.80	0.65	0.19
HPGDS	0.54	0.17	0.24	0.98	0.41	0.79	0.32	0.21
IDO1	0.64	0.18	0.24	0.95	0.59	0.83	0.59	0.17
KLKB1	0.58	0.11	0.33	0.90	0.50	0.72	0.11	0.04
KRAS	0.48	0.14	0.17	0.93	0.38	0.70	0.12	0.03
LTA4H	0.77	0.08	0.57	0.96	0.73	0.89	0.84	0.57
PPARA	0.66	0.11	0.37	0.88	0.58	0.80	0.75	0.34
TLR7	0.58	0.12	0.31	0.83	0.48	0.75	0.33	0.01
TLR8	0.58	0.13	0.30	0.83	0.47	0.77	0.34	0.05
TLR9	0.62	0.11	0.38	0.83	0.54	0.77	0.38	0.01
TRPV3	0.76	0.10	0.41	0.98	0.70	0.87	0.65	0.42
Average	*0.60*	*0.13*	*0.32*	*0.90*	*0.51*	*0.77*	*0.39*	*0.15*
SD	*0.09*	*0.03*	*0.10*	*0.05*	*0.11*	*0.07*	*0.21*	*0.15*

Figure S3. Scatterplot of experimental vs. predicted pACT values for 20 external compounds from the work of Higgins et al.[40] The solid line represents the line of identity bisector, while the dashed lines indicate the model's standard error boundaries. Compound 16 is shown on the right side, with the substructure highlighted in light blue flagged as out-of-applicability.

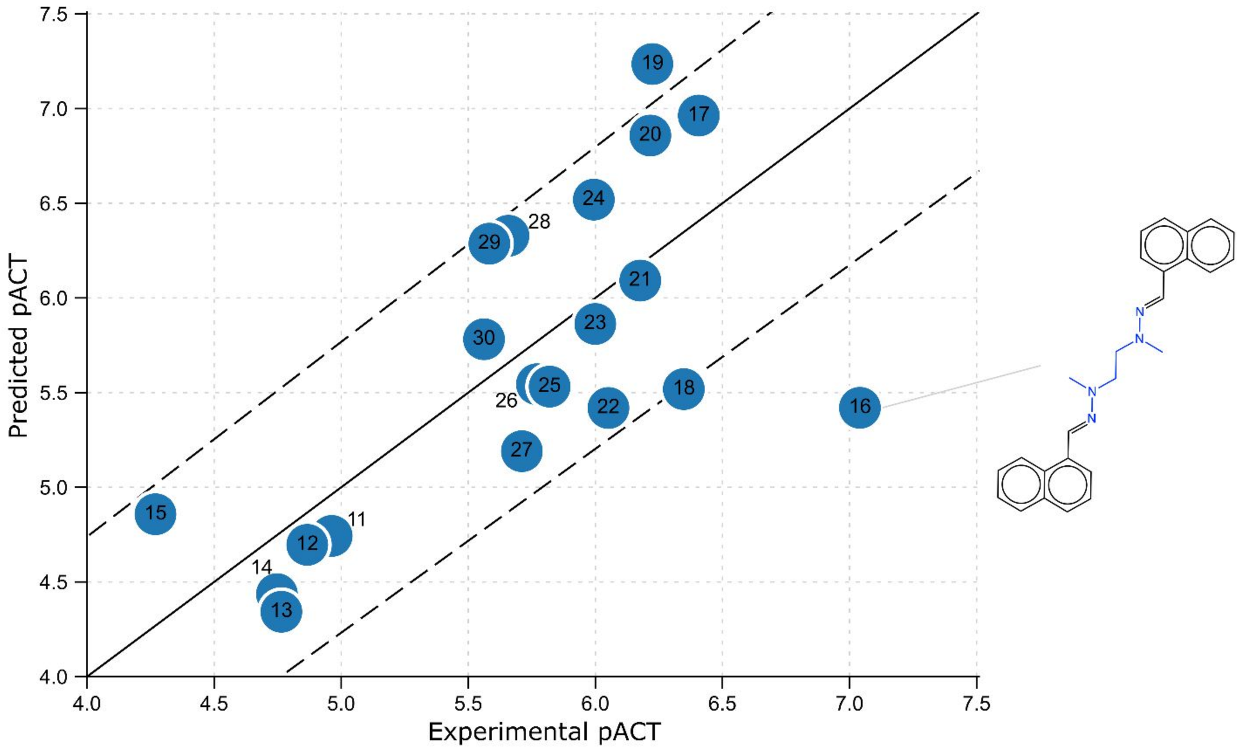

Table S5. List of selected compounds for case study #3.

Index	Smiles	UniProt	Gene Name	Type
1	CC(c1cc(c2cc1)CCN2C(c3cc(Cl)ccc3)=O)NC(c4c(F)cc(Cl)cc4)=O	P14902	IDO1	pIC50
2	Cc1ccc(NC(Nc2c(c3cc(c4c(C(ONC5=O)=N5)cccc4)c2)OCCCC3C6CC6)=O)cc1	P14902	IDO1	pIC50
3	CC(c1cc(c2cc1)CCN2C(C3CCOCC3)=O)NC(c4ccc(F)cc4)=O	P14902	IDO1	pIC50
4	Cc1ccc(NC(Nc2c(c3cc(c4c(c5n[nH]nn5)cccc4)c2)OCC=CC3c6ccccc6)=O)cc1	P14902	IDO1	pIC50
5	CC(CN(c1c(NC(Nc2c(F)cccc2)=O)cc(c3c(C(O)=O)cccc3)cc1)CC(C)C)C	P14902	IDO1	pIC50
6	CC(C(C(C)C)Oc1c(NC(Nc2ccc(C)cc2)=O)cc(c3c(c4[nH]nnn4)cccc3)cc1)C	P14902	IDO1	pIC50
7	NS(N1CC(CNc2c(C(Nc3cc(Br)c(F)cc3)=NO)non2)(O)C1)(=O)=O	P14902	IDO1	pIC50
8	ON=C(c1c(NC(NC2CC2)=O)non1)Nc3cc(Br)c(F)cc3	P14902	IDO1	pIC50
9	ON=C(c1c(NCC2CNCC2)non1)Nc3cc(Br)c(F)cc3	P14902	IDO1	pIC50
10	ON=C(c1c(NCC2CN(S(C3COC3)(=O)=O)CC2)non1)Nc4cc(Br)c(F)cc4	P14902	IDO1	pIC50
11	COc1c(COc2cc(N3C=NN(Cc4n(CC5OCC5)c(c6n4)cc(C(O)=O)cc6F)CC3)ccc2)ccc(Cl)c1	P43220	GLP1R	pEC50
12	CC1c(c2CCN1Cc3n(CC4OCC4)c(c5n3)cc(C(O)=O)cc5F)nc(NCc6c(F)cc(Cl)cc6F)c(C(F)(F)F)c2	P43220	GLP1R	pEC50
13	CCn1c(Cn2c(c3nc2CN4CC(C5(c6nc(OCc7c(OC)cc(C#N)cc7)ccn6)CC4)C5)cc(C(O)=O)cc3OC)cnc1	P43220	GLP1R	pEC50
14	OC(c1c(F)c(c2s1)n(CC3OCC3)c(CN4CC=C(c5nc(OCc6c(c7c(Cl)cc6)oc(F)c7)ccc5)CC4)n2)=O	P43220	GLP1R	pEC50
15	Cc1c(c2ccc(C(O)=O)o2)n(CC3OCC3)c(CN4CCC(c5c(c6ccc5)OC(c7c(F)cc(Cl)cc7)(C)O6)CC4)n1	P43220	GLP1R	pEC50
16	CC1c(c2CCN1Cc3n(CC4OCC4)c(c5n3)nc(C(O)=O)cc5)nc(OCc6c(F)cc(C#N)cc6)c(Cl)c2	P43220	GLP1R	pEC50
17	OC(c1cc(c2cc1)n(C3COCC3)c(CN4N=CN(c5nc(OCc6c(F)cc(C#N)cc6)ccc5)CC4)n2)=O	P43220	GLP1R	pEC50

**18**	OC(c1cc(c2cc1)n(CC3OCC3)c(Cc4c(F)cc(c5nc(OCc6c(c7c(F)cc6)occ7)ccc5)cc4)n2)=O	P43220	GLP1R	pEC50
**19**	OC(c1cc(c2cc1)n(CC3OCC3)c(CN4Cc(c5CC4)cc(OCc6ccc(C(F)F)cc6)c(C(F)(F)F)c5)n2)=O	P43220	GLP1R	pEC50
**20**	OC(c1cc(c2cc1)n(Cc3scnc3)c(CN4CCC(c5nc(N6Cc(c7CC6)c(F)cc(C#N)c7)ccc5)CC4)n2)=O	P43220	GLP1R	pEC50
**21**	Cc1c(n2cc(c3c(c4cc(C#N)ccc4)nc(NC(N5CC6(COC6)C5)=O)s3)c1)ncc2C	P29274	ADORA2A	pKI
**22**	CN1C(C2OCC1)CN(C(Nc3sc(c4cc(c5cc4)c(C)ncn5)c(c6cc(C#N)ccc6)n3)=O)C2	P29274	ADORA2A	pKI
**23**	Cc1nc(C)c(c2c(c3cc(C#N)ccc3)nc(NC(N4CCC(C#N)(C)CC4)=O)s2)o1	P29274	ADORA2A	pKI
**24**	NC(C(N1c(c23)cccc2)=NN(Cc4c(C(F)(F)F)cccc4F)C1=O)=N3	P29274	ADORA2A	pKI
**25**	Nc1c(n2c(c3n1)cccc3)nc(Cc4ccccc4)c2	P29274	ADORA2A	pKI
**26**	COc1cc(c2cc1)N(C(=N3)C(N)=N2)C(=O)N3Cc4ccccc4	P29274	ADORA2A	pKI
**27**	CCCCC#Cc1nc(c2c(N)n1)n(C3C(O)C(O)CO3)c(c4ccco4)n2	P29274	ADORA2A	pKI
**28**	NC(C(N1c(c23)nccc2)=NN(Cc4ccccc4)C1=O)=N3	P29274	ADORA2A	pKI
**29**	Nc1c(n2c(c3n1)cccc3)nc(c4ccccc4)c2	P29274	ADORA2A	pKI
**30**	CCC#Cc1nc(c2c(NCc3cc(I)ccc3)n1)n(C4C(O)C(O)CS4)cn2	P29274	ADORA2A	pKI
**31**	CC(c1c(N)nccc1)N2c(c(c3c(F)c(c4c(C(F)(F)F)c(C)nc(N)c4)n5)c5OCC2)nc(OCC6(CC7(C(F)(F)C7)C8)N8CC6)n3	P01116	KRAS	pIC50
**32**	CC(c1c(N)nccc1)N2c(c(c3nc(c4c(C(F)(F)F)c(C)c(F)c(N)n4)c5Cl)c5OCC2)nc(OCC6(CC(F)C7)N7CCC6)n3	P01116	KRAS	pIC50
**33**	CC(c1c(N)ncs1)N2c(c(c3c(F)c(c4c(C(F)(F)F)c(C)cc(N)n4)n5)c5OCC2)nc(OCC6(CC(F)C7)N7CCC6)n3	P01116	KRAS	pIC50
**34**	CC(c1c(N)nccn1)N2c(c(c3c(F)c(c4c(C(F)(F)F)c(C)c(F)c(N)c4)n5)c5OCC2)nc(OCC6N(C7CC6)CCOC7)n3	P01116	KRAS	pIC50
**35**	CN(C1(CCC1)CNc2c(c3nc(OCC4(CC(=C(F)F)C5)N5CCC4)n2)cnc(c6c(c7cc(O)c6)c(C#C)c(F)cc7)c3F)C	P01116	KRAS	pIC50
**36**	CC1C(N2c(c(c3c(F)c(c4c(c5cnc4)c(C#C)ccc5)n6)c6O1)nc(OCC7(CC(F)C8)N8CCC7)n3)C(NC9C2)CC9	P01116	KRAS	pIC50

**37**	CC(c1c(F)c(C(C(O)(C)C)(F)F)ccc1)Nc2c(c3nc(C)n2)cc(S(C)(=O)=O)nc3C	P01116	KRAS	pIC50
**38**	CCc1c(c2ccc1F)c(c3c(F)c(c4cn3)nc(OCC5(CC5)CN6CCC7(CC(N8CCC(c9cc(c%10cc9)n(C)nc%10)CC8)C7)CC6)nc4N%11CC%12(OCC%12)CCC%11)cc(O)c2	P01116	KRAS	pIC50
**39**	Oc1cc(c2c(c3cc(c4nc3)nc(OCC5(CCC6)N6CCC5)nc4N7CC(NC8C7)CC8)c1)cccc2	P01116	KRAS	pIC50
**40**	CN1C(COc2nc(c3c(N4CC(CC#N)N(C(C=C)=O)CC4)c2)cc(c5c(c6ccc5)c(Cl)ccc6)cn3)CCC1	P01116	KRAS	pIC50
**41**	CC(C(NC1CCN(Cc2ccc(C3Oc(c4OC3)nccc4)cc2)CC1)=O)(O)C	P09960	LTA4H	pIC50
**42**	CN(C1CCCCC1)Cc2ccc(C3Oc(c4OC3)cccc4)cc2	P09960	LTA4H	pIC50
**43**	O=C1N(CC2CN(Cc3ccc(C4Oc(c5OC4)cccc5)cc3)CCC2)CCC1	P09960	LTA4H	pIC50
**44**	O=C1C2(CCN(Cc3ccc(C4Oc(c5OC4)cccc5)cc3)CC2)CCCN1	P09960	LTA4H	pIC50
**45**	C(c1ccc(C2Oc(c3OC2)nccc3)cc1)N4Cc(n5CC4)nnc5	P09960	LTA4H	pIC50
**46**	CC(c1ccc(C2Oc(c3OC2)cccc3)cc1)N4CCC(C(O)=O)CC4	P09960	LTA4H	pIC50
**47**	Cn1ncc(CNCc2ccc(C3Oc(c4OC3)cccc4)cc2)c1	P09960	LTA4H	pIC50
**48**	CCCNCc1ccc(C2Oc(c3OC2)cccc3)cc1	P09960	LTA4H	pIC50
**49**	CCN(Cc1ccc(C(O)=O)cc1)Cc2ccc(C3Oc(c4OC3)cccc4)cc2	P09960	LTA4H	pIC50
**50**	O=C1N(Cc2ccc(C3Oc(c4OC3)nccc4)cc2)CCC1	P09960	LTA4H	pIC50
**51**	COc1c(F)c(CNC(c2c(C3(COC3)F)nn(Cc4ccc(CN5C(=O)C=CC(F)=C5)cc4)c2)=O)c(F)cc1	P03952	KLKB1	pIC50
**52**	COC(Nc1ccc(c2cn(C(c3[n+]([O-])cc(c4c(n5nnnc5)ccc(Cl)c4F)c(OC)c3)CC6CC6)nc2)cc1)=O	P03952	KLKB1	pIC50
**53**	CC(n1nnc(C(NC2CC(c3c(n4ncc(F)c4)ccc(Cl)c3)C2)=O)c1)c5c(C)cc(N6C(=O)C(C7C6)C7)nc5	P03952	KLKB1	pIC50
**54**	[O-][n+]1c(C(n2ncc(c3ccc(C(NC(=O)O4)=N4)cc3)c2)CCOC(F)F)ccc(c5c(n6nnc(C(F)(F)F)c6)ccc(Cl)c5F)c1	P03952	KLKB1	pIC50
**55**	CC(n1nnc(C(NC2CC(c3c(C#N)ccc(Cl)c3)C2)=O)c1)c4c(C)n(C)c(N5C(=O)C(C6C5)C6)n4	P03952	KLKB1	pIC50

**56**	CC(n1ncc(C(NC2CC(c3nccc(C)c3)C2)=O)c1)c4c(C)cc(N5C(=O)C(C6C5)C6)nc4	P03952	KLKB1	pIC50
**57**	CC(C(n1ncc(C(N2CC3(c(c4NC(=O)O3)c(F)c(Cl)cc4)CCC2)=O)c1)c5ccccc5)O	P03952	KLKB1	pIC50
**58**	[O-][n+]1c(C(n2ncc(c3n(C(F)F)ncn3)c2)Cc4nn(C(F)F)cc4)ccc(c5c(n6nnc(C(F)(F)F)c6)ccc(Cl)c5F)c1	P03952	KLKB1	pIC50
**59**	Cn1c(c2cn(C(c3[n+]([O-])cc(c4c(n5c(OC(F)F)cnn5)ccc(Cl)c4F)cc3)CCOC(F)F)nc2)ncn1	P03952	KLKB1	pIC50
**60**	[O-][n+]1c(C(n2ncc(c3nn(C(F)F)cc3)c2)Cc4ccc(F)cc4)ccc(c5c(n6nnnc6)ccc(Cl)c5F)c1	P03952	KLKB1	pIC50
**61**	CC1c(n2c(c3C(c4c(F)cccc4F)=N1)ccc(Cl)c3Cl)nc(C)n2	P31644	GABRA5	pKI
**62**	CC1C(N2c(c3C(c4c(F)cccc4F)=N1)ccc(Cl)c3Cl)=NC(=O)C=C2	P31644	GABRA5	pKI
**63**	COc1c(Cl)cc(C(C=NN(Cc2n(C)nnc2c3nnc(Cl)cc3)C4=O)=C4)cn1	P31644	GABRA5	pKI
**64**	Cc1ncc(c2c(COc3ncc(c4c3)CN(C5CCOCC5)CC4)c(C)on2)cc1	P31644	GABRA5	pKI
**65**	Cn1c(CN2C(=O)C=C(N3CC(OCC(F)F)C3)C=N2)c(c4c(F)cc(C(F)(F)F)cc4)nn1	P31644	GABRA5	pKI
**66**	O=C1c(n2c(c3N1Cc4nccnc4)cccc3)cnc2	P31644	GABRA5	pKI
**67**	CC1OC(C)CN(C(C=NN(Cc2n(c3cnc(C)cc3)nnc2C)C4=O)=C4)C1	P31644	GABRA5	pKI
**68**	Cc1c(COc2ncc(c3c2)CN(c4cnccc4)CC3)n(c5cnc(C(F)(F)F)cc5)nn1	P31644	GABRA5	pKI
**69**	Cc1c(CN2C(=O)C=C(c3cn(C4CC4)nc3)C=N2)n(c5c(F)cc(Cl)cc5)nn1	P31644	GABRA5	pKI
**70**	Cn1c(COc2nnc(C(NN3CCOCC3)=O)cc2)c(c4ccc(F)cc4)nn1	P31644	GABRA5	pKI
**71**	Cc1n(C)ncc1CNC(c2ccc(c3c(c4ccn3)cc[nH]4)cc2)=O	O60760	HPGDS	pIC50
**72**	CC1(CCC(NC(c2cc(F)c(c3c(c4ccn3)cc[nH]4)cc2)=O)CC1)O	O60760	HPGDS	pIC50
**73**	Cn1c(C2CCC(NC(c3cc(F)c(c4c(c5ccn4)cco5)cc3)=O)CC2)nnn1	O60760	HPGDS	pIC50
**74**	Clc1ncnc(N2CC(NC(c3ccc(c4c(c5ccn4)cco5)cc3)=O)CC2)c1	O60760	HPGDS	pIC50

**75**	OC1(N2CCC(NC(c3ccc(c4c(c5ccn4)cco5)cc3)=O)CC2)CC1	O60760	HPGDS	pIC50
**76**	CC(C1CCC(NC(c2ccc(c3c(c4ccn3)nc[nH]4)cc2)=O)CC1)(O)C	O60760	HPGDS	pIC50
**77**	Fc1c(N2CCC(C(N3CCOCC3)=O)CC2)ccc(NC(c4ccc(c5c(c6ccn5)cco6)cc4)=O)c1	O60760	HPGDS	pIC50
**78**	Clc1cc(CNC(c2ccc(c3c(c4ccn3)cco4)cc2)=O)ccc1	O60760	HPGDS	pIC50
**79**	Cc1c(NC(c2ccc(c3c(c4ccn3)cco4)cc2)=O)ccc(S(N)(=O)=O)c1	O60760	HPGDS	pIC50
**80**	Cn1c(c2c(c3c(F)cc(C(NC4CCC(C(O)(C)C)CC4)=O)cc3)n1)ccc(F)c2	O60760	HPGDS	pIC50
**81**	Cc1c(O)ccc(c2c(c3ncc2)ccc(O)c3)c1	Q9NR97	TLR8	pIC50
**82**	CC(c1c(c2[nH]c1C(C3=C(CCC3)C(=O)N4C)=C4)cc(C5CCN(C6COC6)CC5)cc2)C	Q9NR97	TLR8	pIC50
**83**	CC(c1c(c2[nH]c1c3c(c4c(n5c3)ncn5)COC4)cc(C6CCN(CC(NC7CC7)=O)CC6)cc2)C	Q9NR97	TLR8	pIC50
**84**	CC(c1c(c2[nH]c1c3c(c4c(n5c3)ncn5)COC4)cc(C6CCN(C(N7CCN(C)CC7)=O)CC6)cc2)C	Q9NR97	TLR8	pIC50
**85**	CC(c1c(c2[nH]c1c3c(c4c(n5c3)ncn5)COC4)c(F)c(N6CCN(CC(N)=O)CC6)nc2)C	Q9NR97	TLR8	pIC50
**86**	CC(c1c(c2[nH]c1c3c(c4c(n5c3)ncn5)COC4)cc(C6CCN(C(CNS(C)(=O)=O)=O)CC6)cc2)C	Q9NR97	TLR8	pIC50
**87**	CC(c1c(c2[nH]c1c3c(c4c(n5c3)ncn5)COC4)cc(C6CCN(C(CN7CC(F)(F)CC7)=O)CC6)cc2)C	Q9NR97	TLR8	pIC50
**88**	Cc1nc(c2c(c3cc(C)c(O)cc3)c1)cccc2	Q9NR97	TLR8	pIC50
**89**	Cc1c(O)ccc(c2c(c3ncc2)cc[nH]3)c1	Q9NR97	TLR8	pIC50
**90**	COC(c1c(n2nc1)nccc2c3cc(C)ccc3)=O	Q9NR97	TLR8	pIC50
**91**	Cc1c(OC(C(O)=O)(C)C)c(C)cc(CN2C(=O)N(c3ccc(OC(F)(F)F)cc3)CC2=O)c1	Q07869	PPARA	pEC50
**92**	CC(c1ccc(N2C(=O)N(Cc3cc(C)c(OC(C(O)=O)(C)C)c(C)c3)C(=O)C2)cc1)C	Q07869	PPARA	pEC50
**93**	CCc1ccc(N2C(=O)N(Cc3cc(C)c(OC(C(O)=O)(C)C)c(C)c3)C(=O)C2)cc1	Q07869	PPARA	pEC50

**94**	Cc1c(OC(C(O)=O)(C)C)c(C)cc(CN2C(=O)N(c3cc(C(F)(F)F)ccc3)CC2=O)c1	Q07869	PPARA	pEC50
**95**	CC(C(O)=O)(Oc1c(Cl)cc(CN2C(=O)N(c3ccc(C(F)(F)F)cc3)CC2=O)cc1Cl)C	Q07869	PPARA	pEC50
**96**	Cc1c(OC(C(O)=O)(C)C)c(C)cc(CN2C(=O)N(c3ccc(Cl)cc3)CC2=O)c1	Q07869	PPARA	pEC50
**97**	Cc1c(OCc2c(F)cc(F)cc2)ccc(CNc3cc(C(O)=O)ccc3)c1	Q07869	PPARA	pEC50
**98**	Cc1c(OC(C(O)=O)(C)C)c(C)cc(CCCN2C(=O)N(c3ccc(C(F)(F)F)cc3)CC2=O)c1	Q07869	PPARA	pEC50
**99**	Cc1c(c2ccc(C(F)(F)F)cc2)ccc(CNc3cc(C(O)=O)cc(F)c3)c1	Q07869	PPARA	pEC50
**100**	CCc1c(CCC(O)=O)ccc(COc2c(Cc3n(CCN4CCCC4)c(c5n3)cccc5)cccc2)c1	Q07869	PPARA	pEC50
**101**	CC(c1c(c2[nH]c1c3c(c4c(n5c3)ncn5)COC4)cc(C6CCNCC6)cc2)C	Q9NYK1	TLR7	pIC50
**102**	CC(c1c(c2[nH]c1c3c(c4c(n5c3)ncn5)COC4)cc(C6CCN(C(N7CCN(C)CC7)=O)CC6)cc2)C	Q9NYK1	TLR7	pIC50
**103**	CC(c1c(c2[nH]c1c3c(c4c(n5c3)ncn5)COC4)cc(C6CCN(C(CNC7CC7)=O)CC6)cc2)C	Q9NYK1	TLR7	pIC50
**104**	CC(c1c(c2[nH]c1C(C3=C(CCC3)C(=O)N4C)=C4)cc(C5CCN(C6COC6)CC5)cc2)C	Q9NYK1	TLR7	pIC50
**105**	CC(c1c(c2[nH]c1c3c(c4c(n5c3)ncn5)COC4)cc(C6CCN(CC(NC7CC7)=O)CC6)cc2)C	Q9NYK1	TLR7	pIC50
**106**	CC(c1c(c2[nH]c1c3c(c4c(n5c3)ncn5)COC4)cc(C6CCN(CCS(N)(=O)=O)CC6)cc2)C	Q9NYK1	TLR7	pIC50
**107**	CC(c1c(c2[nH]c1c3c(c4c(n5c3)ncn5)COC4)cc(C6CCN(C(CN7CC(O)CC7)=O)CC6)cc2)C	Q9NYK1	TLR7	pIC50
**108**	CC(c1c(c2[nH]c1c3c(c4c(n5c3)ncn5)COC4)cc(C6CCN(CC(N7CC(F)(F)CC7)=O)CC6)cc2)C	Q9NYK1	TLR7	pIC50
**109**	CC(c1c(c2[nH]c1c3c(c4c(n5c3)ncn5)COC4)cc(C6CCN(CC7(C#N)CC7)CC6)cc2)C	Q9NYK1	TLR7	pIC50
**110**	NC1CN(c2c(c3c(C#N)cc2)cccn3)CC(C(F)(F)F)C1	Q9NYK1	TLR7	pIC50
**111**	CC1N(c2c(OC3CN(c4c5c(c(c6o5)cccc6)nc(C(F)F)n4)C(C(O)=O)C3)ncc(Br)c2)CCN(C#N)C1	Q8N884	CGAS	pIC50
**112**	CC1N(c2c(OC3CN(c4c5c(c(c6o5)cccc6)nc(C)n4)C(C(O)=O)C3)ncc(C#C)c2)CCOC1	Q8N884	CGAS	pIC50

**113**	OC(C1N(c2c3c(c(c4o3)cccc4)nc(C(F)(F)F)n2)CC(Oc5c(N6Cc(c7CC6)con7)cc(Cl)cn5)C1)=O	Q8N884	CGAS	pIC50
**114**	CC1N(c2c(OC3CN(c4c5c(c(c6o5)cccc6)nc(C(F)(F)F)n4)C(C(O)=O)C3)ncc(c7cn(C)nc7)c2)CCOC1	Q8N884	CGAS	pIC50
**115**	CC1C2(COC2)OCCN1c3c(OC4CN(c5c6c(c(c7o6)cccc7)nc(C(F)(F)F)n5)C(C(O)=O)C4)ncc(c8c(C#N)nccc8)c3	Q8N884	CGAS	pIC50
**116**	Sc1[nH]c(c2c(c3c[nH]nc3)c(c4[nH]2)ccc(Cl)c4Cl)nn1	Q8N884	CGAS	pIC50
**117**	CC1c(c2CCN1C(CO)=O)c(c3[nH]2)ccc(Cl)c3Cl	Q8N884	CGAS	pIC50
**118**	COc1ncc(F)c(c2cc(c3nc2)C(C(CN4C(=O)CCCOCCc5nc(c6c(c(c7o6)cccc7)n5)N(CC8O3)C(C(O)=O)C8)C)(F)CC4)c1	Q8N884	CGAS	pIC50
**119**	COC(C1N(c2c3c(c(c4o3)cccc4)nc(C(F)F)n2)CC(Oc5c(C6(C(C)CS(=O)(=O)CC6)F)cc(Cl)cn5)C1)=O	Q8N884	CGAS	pIC50
**120**	Cn1nc(c2c(c3c(Cl)c(Cl)c2)c(n4n3)CN(C(CO)=O)CC4)cc1	Q8N884	CGAS	pIC50
**121**	Fc1cc(NC(c2nc(n3cncc3)ncc2)=O)cc(F)c1	P28907	CD38	pIC50
**122**	FC(c1cc(n2cncc2)nc(C(Nc3cnccc3)=O)c1)(F)F	P28907	CD38	pIC50
**123**	FC(c1nc(n2cncc2)nc(C(Nc3cnncc3)=O)c1)(F)F	P28907	CD38	pIC50
**124**	COCCOc1ncc(NC(c2nc(n3cncc3)nc(C(F)(F)F)c2)=O)cn1	P28907	CD38	pIC50
**125**	COC1CCC(NC(c2nc(n3cncc3)nc(OC)c2)=O)CC1	P28907	CD38	pIC50
**126**	COC1CCC(NC(c2nc(n3cncc3)cnc2)=O)CC1	P28907	CD38	pIC50
**127**	O=C(c1nc(n2cncc2)cnc1)Nc3cnccc3	P28907	CD38	pIC50
**128**	CC1CCC(NC(c2nc(n3cncc3)nc(C(F)(F)F)c2)=O)CC1	P28907	CD38	pIC50
**129**	COC1CCC(NC(c2nc(n3cncc3)cc(N4CCCC4)c2)=O)CC1	P28907	CD38	pIC50
**130**	FC(c1nccc(NC(c2nc(n3cncc3)ccc2)=O)c1)(F)F	P28907	CD38	pIC50
**131**	ONC(c1ccc(Cn2nc(c3c(F)cccc3)nc2)cc1)=O	Q9UBN7	HDAC6	pIC50

**132**	FC(c1nnc(c2cnc(Cn3nnc(c4cc(c5nc4)cc[nH]5)c3)cc2)o1)F	Q9UBN7	HDAC6	pIC50
**133**	FC(c1nnc(c2ccc(Cn3nnc(c4ccc(NC(NCC5)=N5)cc4)c3)cc2)o1)F	Q9UBN7	HDAC6	pIC50
**134**	ONC(c1ncc(NC(C(c2ccccc2)Cc3ccccc3)=O)cc1)=O	Q9UBN7	HDAC6	pIC50
**135**	FC(c1nnc(c2cnc(CN(S(N3CCS(=O)(=N)CC3)(=O)=O)c4ccccc4)cc2)o1)F	Q9UBN7	HDAC6	pIC50
**136**	FC(c1nnc(c2cnc(Cn3nc(c4ccccc4)nn3)cc2)o1)F	Q9UBN7	HDAC6	pIC50
**137**	CC(n1nnc(c2cc(c3cc2)sc(N)n3)c1)c4c(F)cc(c5nnc(C(F)F)o5)cc4	Q9UBN7	HDAC6	pIC50
**138**	FC(c1nnc(c2cc(F)c(CN(c3cc(c4cc3)CCO4)C(N5CCS(=O)(=N)CC5)=O)cc2)o1)F	Q9UBN7	HDAC6	pIC50
**139**	ONC(c1ccc(C=CC(N2CC(CNC3C(c4ccc(F)cc4)C3)C2)=O)cc1)=O	Q9UBN7	HDAC6	pIC50
**140**	ONC(c1ccc(CCCN2CCC(CNC3C(c4cn(c5ccccc5)nc4)C3)CC2)cc1)=O	Q9UBN7	HDAC6	pIC50
**141**	COC1C(C2C(C)(C3C1)CCC(O)C3)C(C4(C)CC2)CCC4C(CCC(N5CCCC5)=O)C	Q8TDU6	GPBAR1	pEC50
**142**	COc1c(c2c(N(C(c3cc(S(N(C)C)(=O)=O)cc(C(F)(F)F)c3)=O)C)cncc2)ccc(F)c1	Q8TDU6	GPBAR1	pEC50
**143**	CN(c1c(c2c(F)cccc2F)ccnc1)C(c3cc(C(F)(F)F)cc(C(F)(F)F)c3)=O	Q8TDU6	GPBAR1	pEC50
**144**	COc1c(c2c(N(C(c3cc(S(C)(=O)=O)cc(C(F)(F)F)c3)=O)C)cncc2)cccc1	Q8TDU6	GPBAR1	pEC50
**145**	CN(c1c(c2c(C)cccc2)ccnc1)C(c3cc(C(F)(F)F)nc(C(F)(F)F)c3)=O	Q8TDU6	GPBAR1	pEC50
**146**	CCc1c(c2c(N(C(c3cc(C(F)(F)F)cc(C(F)(F)F)c3)=O)C)cncc2)cccc1	Q8TDU6	GPBAR1	pEC50
**147**	COc1c(c2c(N(C(c3cc(S(C)(=O)=O)nc(C(F)(F)F)c3)=O)C)cncc2)ccc(F)c1	Q8TDU6	GPBAR1	pEC50
**148**	CN(c1c(c2c(C)cccc2)cc(C)nc1)C(c3cc(C(F)(F)F)cc(C(F)(F)F)c3)=O	Q8TDU6	GPBAR1	pEC50
**149**	COc1c(c2c(N(C(c3cc(S(C)(=O)=O)cc(C(F)(F)F)c3)=O)C)c(Cl)ncc2)ccc(F)c1	Q8TDU6	GPBAR1	pEC50
**150**	CN(c1c(c2c(OCC(F)(F)F)nccc2)ccnc1)C(c3cc(S(C)(=O)=O)cc(C(F)(F)F)c3)=O	Q8TDU6	GPBAR1	pEC50

**151**	COc1c(OC)cc(c2n(C)c(c3n2)cc(c4ccc(N5CC6(CN(C7CCC7)C6)C5)cc4)nc3C)cc1	Q9NR96	TLR9	pIC50
**152**	CC(CN1CC(C2C1)CN(c3ccc(c4nc(C)c5c(n(C)c(c6ccc(S(C)(=O)=O)cc6)n5)c4)cc3)C2)C	Q9NR96	TLR9	pIC50
**153**	CC(CN1CCC(N2CCC(c3nc(C)c4c(n(C)c(c5ccc(S(C)(=O)=O)cc5)n4)c3)CC2)CC1)C	Q9NR96	TLR9	pIC50
**154**	Cc1c2c(n(C)c(c3ccc(S(C)(=O)=O)cc3)n2)cc(c4ccc(N5CCC6(CN(C7CCC7)C6)CC5)cc4)n1	Q9NR96	TLR9	pIC50
**155**	Cc1c2c(n(C)c(c3ccc(S(C)(=O)=O)cc3)n2)nc(C4CCN(C5CCN(C6CCOCC6)CC5)CC4)c1	Q9NR96	TLR9	pIC50
**156**	CC(CN1CC2(CN(Cc3ccc(c4nc(c5c(C)c4)n(C6CC6)c(c7ccc(S(C)(=O)=O)cc7)n5)cc3)C2)C1)C	Q9NR96	TLR9	pIC50
**157**	COCCN1CC(C2C1)CC(N3CCC(c4nc(c5c(C)c4)n(C)c(c6cc(OC)c(OC)cc6)n5)CC3)C2	Q9NR96	TLR9	pIC50
**158**	CN(C1CCN(Cc2ccc(c3nc(C)c4c(n(C)c(c5ccc(S(C)(=O)=O)cc5)n4)c3)cc2)CC1)C	Q9NR96	TLR9	pIC50
**159**	COc1c(OC)cc(c2[nH]c(c3n2)cc(C4CCN(C5CCN(CC(C)C)CC5)CC4)cn3)cc1	Q9NR96	TLR9	pIC50
**160**	COc1c(n2cc(c3n(C)c(c4n3)nc(C5CCN(C6CC(C7C6)CN(C8CCC8)C7)CC5)cc4C)c1)ncn2	Q9NR96	TLR9	pIC50
**161**	CC(c1ccc(C(C2(CN(C)C2)C)(c3cc(c4nc(COc5cn(C)nc5)no4)ccc3)O)cc1)C	P51684	CCR6	pIC50
**162**	CC(c1ccc(C(C2(CN(C)C2)C)(c3cc(C#CC(c4ncnc(C)c4)(O)C)ccc3)O)cc1)C	P51684	CCR6	pIC50
**163**	CC(c1ccc(C(C2(CN(C)C2)F)(c3cc(c4nc(C5CCN(C(C)=O)CC5)on4)ccc3)O)cc1)C	P51684	CCR6	pIC50
**164**	CC(c1ccc(C(C2(CN(C)C2)C)(c3cc(c4nc(C5C(OC6C5)CC6)no4)ccc3)O)cc1)C	P51684	CCR6	pIC50
**165**	CC(c1ccc(C(C2(CN(C)C2)C)(c3cc(c4nc(c5noc(C)c5)no4)ccc3)O)cc1)C	P51684	CCR6	pIC50
**166**	CC(c1ccc(C(C2(CN(C)C2)C)(c3cc(c4nc(C5(CC6(NC(C)=O)C5)C6)on4)cnc3)O)cc1)C	P51684	CCR6	pIC50
**167**	CN1CC(C(c2cc(n3nccc3)ccc2)(c4ccc(OC(F)(F)F)cc4)O)(C)C1	P51684	CCR6	pIC50
**168**	CN1CC(C(c2cc(N3CCCC3)ccc2)(c4ccc(OC(F)(F)F)cc4)O)(C)C1	P51684	CCR6	pIC50
**169**	CN1CC(C(c2ccc(OC(F)(F)F)cc2)(c3ccc(Oc4cnccc4)cc3)O)(C)C1	P51684	CCR6	pIC50

**170**	CC(c1ccc(C(C2(CN(C)C2)C)(c3cc(c4nn(C5CC5)c(C6CCC(O)CC6)n4)cnc3)O)cc1)C	P51684	CCR6	pIC50

Table S6. Comparative overview of ProfhEX and other publicly available drug–target prediction tools. Columns report the ML model type, training data source, dataset size, performance metrics, number of modeled targets, distinctive features, and web access URL.

Tool	ML Model Type	Training Data Source	Data Size	Performance Metrics	Number of Targets	Key Features	URL
**ProfhEX**	Random Forest, Gradient Boosting, Feed-forward neural networks (MLPs)	ChEMBL, PubChem, GOSTAR	>5 million bioactivity data points	r ≥ 0.7, R^2 ≥ 0.5, RMSE ≤ 20% of target range	969 models spanning 693 human targets	Enhanced version with comprehensive target coverage; MLOps automation	https://profhex.exscalate.eu/
**PASS Targets**	Modified Naïve Bayes	ChEMBL v19	589,107 chemical compounds, 2,507 protein targets	AUC ROC ~96% (cross-validation), ~90% (external test)	2,507 protein targets	Multi-target predictions; structure-activity relationships	http://www.way2drug.com/PASSOnline
**AmIActive (AIA)**	Random Forest	ChEMBL 30	3,239 models from 2,277 distinct targets	Average MCC 0.65 (MCC ≥0.5 for model selection); Average accuracy 82%	2,277 distinct targets (single proteins, protein complexes, families, cell lines,	ECFP4 fingerprints; applicability domain calculation	https://amiactive.ccen.ufpb.br/

					organisms, tissues)		
**TargetNet**	Naïve Bayes	BindingDB	109,061 compounds, 115,257 activity endpoints	AUC scores 75-100%;	623 human proteins	Multi-target SAR methodology; ensemble learning with 7 fingerprint types (FP2, Daylight-like, MACCS, Estate, ECFP2, ECPF4 and ECFP6)	http://targetnet.scbdd.com
**CODD-Pred**	Double Molecular Graph Perception (DMGP): TrimNet + DMPNN for target prediction; Multi-model Self-validation Activity Prediction (MSAP) for bioactivity prediction	GOSTAR database	646,498 molecules with 640 human targets	Top-5 accuracy >80%; R^2 > 0.6 for bioactivity models (125 models with $R^2 \geq$ 0.8, 109 models with R^2 values between 0.7 and 0.8, and only a few	640 human targets (target prediction) + 56 disease-related targets (bioactivity)	Dual-purpose: target identification + bioactivity prediction	http://codd.iddd.group/

				models with R2 values between 0.6 and 0.7)			
**SwissTargetPrediction**	Multiple logistic regression to weight 2D and 3D similarity parameters for a dual-scoring, ligand-based reverse screening approach	ChEMBL v23	376,342 unique compounds, 580,496 binding activities	>70% success rate (correct target in top-15 predictions for external compounds)	3,068 targets (2,092 human, 535 rat, 441 mouse)	2D (FP2 fingerprints) + 3D (ES5D vectors) similarity; Combined-Score weighting; fast processing (15-20s); homology-based predictions	https://www.swisstargetprediction.ch
**SuperPred 3.0**	Logistic regression performed best for ATC; various ML models for targets (logistic regression, linear discrimina	ChEMBL v29 (targets), WHO ATC database (drug classification)	365,719 strong binding substances, 500,979 unique relations, 2,353 targets	82% of target models achieve ≥85% accuracy; ATC prediction: 70.1% accuracy	691 targets with ≥20 binders and non-binders	Dual functionality: ATC classification and target prediction; includes non-binders in training; Morgan fingerprints	https://prediction.charite.de/index.php

	nt analysis, k-nearest neighbors, decision tree, support vector machines, gaussian naïve bayes and random forests)						

Table S7. Performance of each tool in identifying correct primary targets after in-domain filtering. The table shows the average percentage of correct primary targets identified within the top-1, top-5, top-10, top-15, and top-20 predictions, considering only compounds from case study #3 whose primary target fell within the applicability domain (AD). Column (*) indicates the number of targets included after the AD check.

**Tool**	**Top N accuracy (%)**					*** N° of included targets**
	**N = 1**	**N = 5**	**N = 10**	**N = 15**	**N = 20**	
**ProfhEX AD All**	21	36	42	52	58	17
**ProfhEX AD In**	**54**	**85**	**91**	**95**	**98**	12
**SwissTargetPrediction**	18	35	43	48	48	11
**SuperPred**	14	32	45	49	49	8
**PASS**	0	3	11	30	41	5
**CODD-Pred**	48	66	71	75	76	10
**TargetNet**	0	8	18	28	40	6
**AmlActive**	9	30	38	42	48	4

Case study 4. Virtual screening performance evaluation on the LIT-PCBA benchmark dataset

LIT-PCBA is a curated benchmark dataset for virtual screening, derived from dose–response bioassays.[2] It is specifically designed to minimize chemical bias and assay artifacts, making it suitable for evaluating both ligand-based and structure-based computational methods. From the 15 targets included in LIT-PCBA, we performed virtual screening analyses on 12 targets, excluding FEN1, KAT2A, and GBA due to the lack of validated ProfhEX models for these receptors. Raw data are available in our Zenodo repository (Case Study #4).

The evaluation of ProfhEX models on LIT-PCBA shows variable performance across targets, best captured by enrichment factor at 1% (EF 1%) and ROC AUC metrics (**Table S8**). Across most targets, EF values improved when predictions were restricted to compounds within the applicability domain (AD), compared to both the baseline KNN similarity search and the full compound set. For example, PPARG and PKM2 exhibit substantial EF gains (29.13 and 8.97, respectively) under AD filtering, well above their baseline values (16.64 and 0.74). This demonstrates that AD filtering can enhance early recognition of actives. However, this improvement comes at the cost of reduced chemical diversity: AD filtering favors compounds structurally closer to the training data, which limits scaffold novelty. To quantify this effect, we compared the average Tanimoto similarity of the top 1% ranked compounds in the full set versus the AD-restricted subset. As expected, the average similarity increased from 0.45 (all compounds) to 0.71 (AD only), reflecting a strong prioritization of compounds resembling the training data and highlighting the trade-off between precision and novelty. This is a well-known limitation of QSAR-based approaches.

This trade-off was particularly evident for ESR1_ago and TP53, where EF dropped or disappeared under AD constraints, suggesting that some actives of these targets lay outside the model's learned chemical space. ROC AUC values complemented this picture by reflecting global ranking ability. In some cases, such as MAPK1, ROC AUC remains moderate (0.55–0.58) despite low EF, indicating that the model ranked actives reasonably well overall, even if early enrichment was weak. Conversely, targets like OPRK1, PKM2, and PPARG show concurrent improvements in both EF and ROC AUC under AD filtering, reinforcing confidence in predictions for these targets. Interestingly, for OPRK1 and PPARG, the baseline 2D similarity search achieved very high EF values (16.67 and 16.64, respectively), even outperforming ProfhEX on the full compound set. This suggests that for certain targets, simple similarity-based approaches can already be highly effective, and ProfhEX provides additional benefit mainly when coupled with AD filtering. To mitigate this drawback and better assess model reliability, additional metrics such as uncertainty estimation should be considered in future iterations. Finally, the relatively high standard deviation of EF values across targets underscores the variability inherent in early enrichment, driven by differences in dataset size, target biology, and chemical distribution. Such variability is typical in prospective virtual screening benchmarks. Overall, ProfhEX achieved average EF%1 values above the baseline and performed best under AD filtering, indicating that it can extract useful structure–activity patterns from a chemically unbiased dataset such as LIT-PCBA, while also highlighting the balance between accuracy and scaffold novelty.

Table S8. Overview of virtual screening performance across 12 LIT-PCBA targets using ProfhEX models. The table reports the number of compounds and actives per target, along with enrichment factor at 1% (EF 1%) and ROC AUC values under three conditions: 2D similarity search taken from the original publication (Baseline), ProfhEX predictions on all compounds (All data), and predictions restricted to compounds within the applicability domain (In AD). Summary statistics (average, standard deviation, median, and median absolute deviation) are provided for each metric.

Target	# of compounds	# of actives	EF 1%			ROC AUC	
			Baseline	All data	In AD	All data	In AD
**ADRB2**	311765	17	0.00	17.65	11.76	0.62	0.55
**ALDH1**	107237	5363	1.58	1.84	1.47	0.58	0.56
**ESR1_ago**	4391	13	0.00	15.35	7.68	0.76	0.70
**ESR1_ant**	3908	88	2.67	2.22	5.55	0.61	0.48
**IDH1**	358796	39	1.59	2.56	5.13	0.66	0.61
**MAPK1**	61875	308	0.95	0.32	2.92	0.55	0.58
**MTORC1**	33069	97	0.00	0.00	1.03	0.46	0.50
**OPRK1**	269499	24	16.67	4.17	9.66	0.46	0.67
**PKM2**	245225	546	0.74	1.10	8.97	0.53	0.63
**PPARG**	4095	24	16.64	8.32	29.13	0.61	0.68
**TP53**	3409	64	0.00	3.04	0.00	0.52	0.47
**VDR**	263303	655	3.64	1.22	1.68	0.52	0.45
		Average	*3.71*	*4.82*	*7.08*	*0.57*	*0.57*
		(SD)	*(5.9)*	*(5.6)*	*(7.6)*	*(0.08)*	*(0.08)*
		Median	*1.27*	*2.39*	*5.34*	*0.56*	*0.56*
		(MAD)	*(1.3)*	*(1.5)*	*(3.8)*	*(0.05)*	*(0.08)*

Supporting References

(1) Lunghini, F.; Fava, A.; Pisapia, V.; Sacco, F.; Iaconis, D.; Beccari, A. R. ProfhEX: AI-Based Platform for Small Molecules Liability Profiling. *J Cheminform* **2023**, *15*, 60.

(2) Tran-Nguyen, V.-K.; Jacquemard, C.; Rognan, D. LIT-PCBA: An Unbiased Data Set for Machine Learning and Virtual Screening. *J Chem Inf Model* **2020**, *60*, 4263–4273.
